# Hybrid Electro‐optical Stimulation Improves Ischemic Brain Damage by Augmenting the Glymphatic System

**DOI:** 10.1002/advs.202417449

**Published:** 2025-02-10

**Authors:** Min Jae Kim, Jiman Youn, Hong Ju Lee, Seo‐Yeon Lee, Tae‐Gyu Kim, Young‐Jin Jung, Yong‐Il Shin, Byung Tae Choi, Joonsoo Jeong, Hwa Kyoung Shin

**Affiliations:** ^1^ Department of Korean Medical Science School of Korean Medicine Pusan National University Yangsan Gyeongnam 50612 Republic of Korea; ^2^ Graduate Training Program of Korean Medical Therapeutics for Healthy‐Aging Pusan National University Yangsan Gyeongnam 50612 Republic of Korea; ^3^ Department of Information Convergence Engineering Pusan National University Yangsan 50612 Republic of Korea; ^4^ Department of Pharmacology Wonkwang University School of Medicine Iksan 54538 Republic of Korea; ^5^ School of Healthcare and Biomedical Engineering Chonnam National University Yeosu 59626 Republic of Korea; ^6^ Department of Rehabilitation Medicine School of Medicine Pusan National University Yangsan Gyeongnam 50612 Republic of Korea; ^7^ School of Biomedical Convergence Engineering Pusan National University Yangsan 50612 Republic of Korea

**Keywords:** aquaporin‐4 polarization, cerebrospinal fluid, glymphatic system, hybrid electro‐optical stimulation, ischemic stroke

## Abstract

Ischemic brain injury not only results in significant neurological, motor, and cognitive impairment but also contributes to the accumulation of toxic solutes and proinflammatory cytokines in the infarction region, exacerbating ischemic brain damage. The glymphatic system, which is crucial for brain waste clearance and homeostasis, is impaired by ischemic injury, highlighting the importance of developing therapeutic strategies for poststroke complications. Herein, a novel hybrid electro‐optical stimulation device is proposed that integrates near‐infrared micro‐light‐emitting diode with transparent microneedles, enabling efficient noninvasive stimulation of the cortical area for ischemic stroke treatment. This study investigates whether this hybrid electro‐optical stimulation enhances the glymphatic system function and ameliorates ischemic brain injury in the middle cerebral artery occlusion and reperfusion (MCAO/R) mice model. The results demonstrate that hybrid stimulation improves the neurological, motor, and cognitive functions and reduces brain atrophy following MCAO/R. Moreover, hybrid stimulation restores impaired glymphatic system function by modulation of aquaporin‐4 (AQP4) polarization and alleviates the accumulation of proinflammatory cytokines such as IL‐1β. Notably, AQP4 inhibition partly reverses the improved functional outcomes of hybrid stimulation. The findings suggest that targeting glymphatic drainage using hybrid electro‐optical stimulation is a promising therapeutic approach for treating ischemic brain injury.

## Graphical abstract

A device is developed that allows simultaneous electrical and optical stimulations. The stimulator modulates AQP4 polarization, which is essential for enhancing glymphatic clearance, thus improving brain recovery from ischemic damage and reducing neurological, motor, and cognitive deficits. This dual‐modality approach not only lessens the impact of ischemic injury, but also supports enhanced recovery and redefines therapeutic interventions for brain injuries.

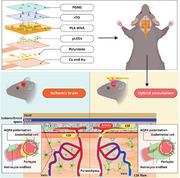

## 1 Introduction

Ischemic brain conditions present significant challenges in neurological medicine, often resulting in profound consequences due to compromised blood flow to the brain.^[^ ^1^ ^]^ Despite the declining stroke mortality rates in recent decades, many survivors still experience various motor, cognitive, and psychiatric impairments.^[^ ^2^ ^]^ The regions affected by infarction contain elements linked to neurotoxicity, neuroinflammation, and neurodegeneration, which worsen tissue and functional impairment and correlate with stroke severity.^[^ ^3^ ^]^ Recent studies have suggested that the early stages of ischemic stroke can activate peripheral immune responses through the drainage of cerebrospinal fluid (CSF) containing dissolved proinflammatory cytokines, thereby worsening the brain injury.^[^ ^4^ ^]^ In addition, the disruption of tau protein clearance by the glymphatic system may exacerbate poststroke cognitive decline, potentially leading to poststroke dementia.^[^ ^5^ ^]^ Hence, controlling the glymphatic system is crucial in the treatment of ischemic stroke.

Previous studies have reported the significant role of persistent impairment in the perivascular drainage of the glymphatic system in various central nervous system (CNS) disorders, such as traumatic brain injury, Alzheimer's disease (AD), Parkinson's disease, and stroke.^[^ ^6^ ^]^ The glymphatic system in the brain facilitates the clearance of metabolic waste by enabling CSF to flow from the para‐arterial influx to the para‐venular efflux, along with various solutes.^[^ ^7^ ^]^ This process involves the exchange of CSF with the interstitial fluid (ISF) in the brain parenchyma. Further studies have revealed the crucial role of aquaporin‐4 (AQP4) polarization in astrocytic endfeet within the glymphatic system.^[^ ^8^ ^]^ Mestre et al. demonstrated a significant reduction in CSF tracer transport in AQP4‐knockout (KO) mice and rats compared with controls.^[^ ^8a^ ^]^ Moreover, ischemic stroke impaired the glymphatic clearance and AQP4 depolarization 2 d after the insult, yet the pharmacological enhancement of glymphatic system function alleviated edema and improved cognitive function. Glymphatic dysfunction in patients with ischemic stroke correlates with the severity of brain edema and overall outcome.^[^ ^9^ ^]^ Glymphatic system dysfunction contributes to stroke risk and is implicated in multiple aspects of stroke pathology, including brain edema, blood‐brain barrier disruption, immune cell infiltration, neuroinflammation, and neuronal apoptosis.^[^ ^10^ ^]^ Hence, targeting the glymphatic system has a therapeutic potential for ischemic stroke management.

Among emerging approaches, neurostimulation has garnered considerable attention because of its potential to enhance stroke recovery by modulating neural activity and plasticity.^[^ ^11^ ^]^ Both electrical and optical stimulation have shown promise in promoting neural plasticity and functional recovery poststroke.^[^ ^12^ ^]^ Electrical stimulation (ES) improves motor and cognitive functions, as well as rehabilitation outcomes, after ischemic stroke, by modulating the neural networks and potentially restoring the lost functionality.^[^ ^13^ ^]^ Optical stimulation (OS) has been shown to inhibit neuroinflammation and increase neuronal survival, thereby improving neurological, motor, and cognitive function poststroke.^[^ ^14^ ^]^ However, each method has limitations, including restricted spatial precision and limited tissue penetration. To overcome these challenges and capitalize on the unique strengths of both modalities, we propose a novel hybrid stimulator that combines electrical and OSs to maximize the therapeutic efficacy and minimize the adverse effects. This approach uses a new transparent and conductive microneedle array (MNA) that enhances the spatial focality and integrates both stimulations. Our in vivo experiments assessed the efficacy of this hybrid stimulation in reducing ischemic brain damage and improving functional outcomes by modulating glymphatic dysfunction and AQP4 polarization. This innovative method can significantly advance stroke rehabilitation and improve the quality of life of stroke survivors.

## 2 Results

### 2.1 Concept of Implantable Hybrid Electro‐optical Stimulator Function for Mouse Model Studies

**Figure 1:**
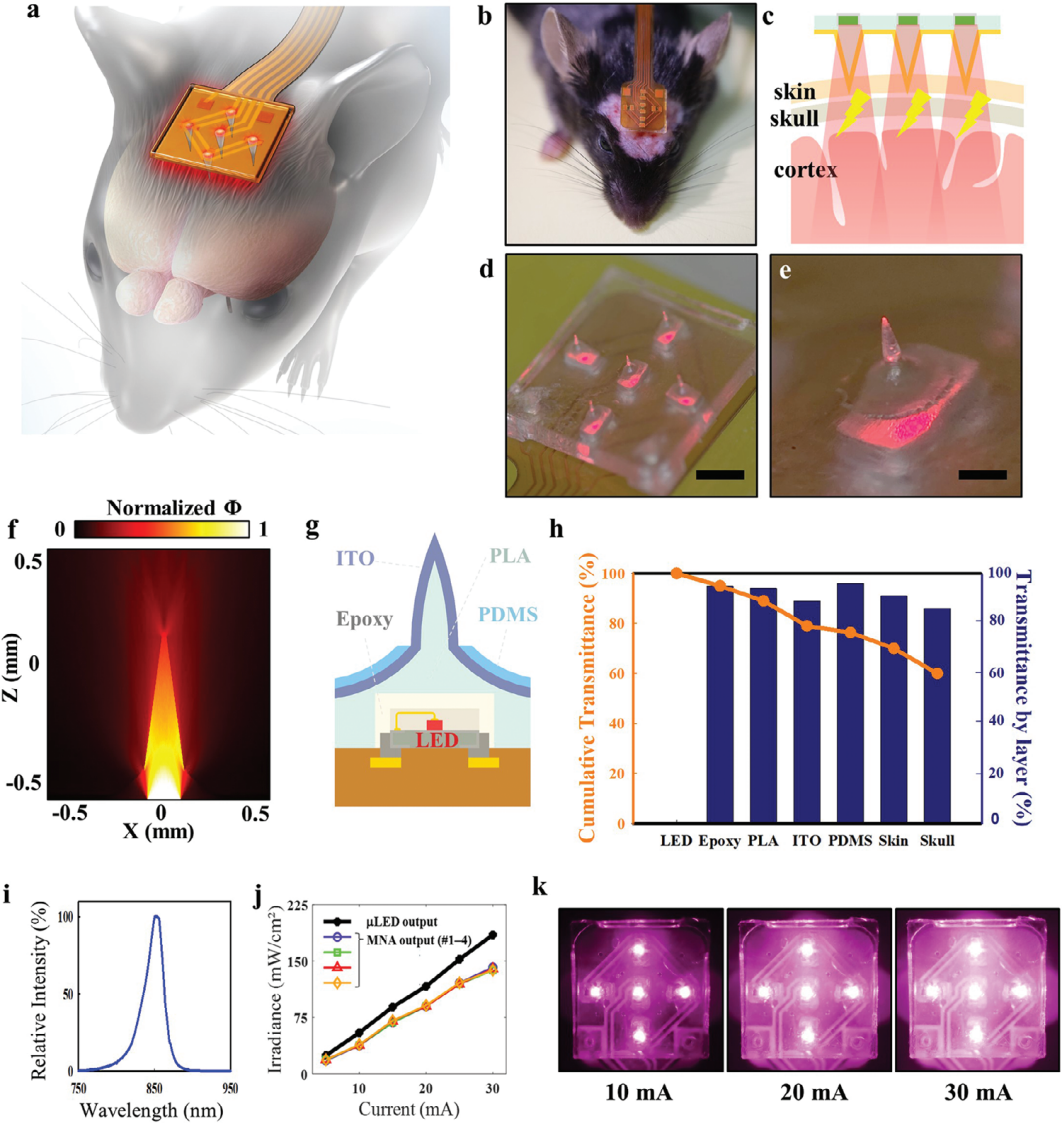
Concept and optical characterization of hybrid electro‐optical stimulator based on a transparent conductive microneedle array (MNA) for stroke rehabilitation in a mouse model. a) Illustration and b) photograph of the device and animal for hybrid electro‐optical stimulation. c) Cross‐sectional presentation of hybrid electro‐optical stimulation. d) Photograph of light emitted through a PLA MNA and e) a magnified view of a single needle (630 nm µLEDs were used here for visual demonstration of optical emission). Scale bar: 2 mm (d) and 0.5 mm (e). f) Monte Carlo simulation of the spatial distribution of normalized light intensity (Φ) from a µLED underneath PLA microneedle. g) Cross‐sectional structure of a PLA microneedle consisting of a µLED pocket, conductive ITO layer, and insulating PDMS layer. h) Optical transmittance (850 nm) of each layer between µLED and cortex, and cumulative transmittance from µLED to cortex. i) Emission spectrum of 850 nm µLED. j) Irradiance of light emitted from a µLED and four PLA MLA devices. k) Photographs showing increasing light intensity with varying driving currents from F, 10 to 30 mA (750 nm IR filter was used to visualize the 850 nm light). Abbreviations: Polydimethylsiloxane (PDMS), polylactide (PLA), and indium tin oxide (ITO).

**Figure** **1** shows the concept and optical characterization of the hybrid electro‐optical stimulator based on a transparent and conductive MNA for stroke rehabilitation in a mouse model. As shown in Figure 1a,b, the stimulator was mounted on the scalp of the mouse to deliver hybrid electro‐optical stimulation to the cortical area beneath the bregma (Figure 1c). This minimally invasive approach reduces surgical complexity and enables synergistic therapeutic effectiveness through simultaneous photobiomodulation (PBM) and transcranial direct current stimulation (tDCS). The micro light‐emitting diodes (µLEDs) for near‐infrared (850 nm) were mounted on a flexible printed circuit board (FPCB) to deliver electrical and optical stimulation through a transparent polyactide (PLA) MNA coated with an electrically conductive indium tin oxide (ITO) layer, as shown in Figure 1d,e (630 nm was used for visualization of light emission). The PLA MNA measured 6 × 6 mm and carried five microneedles, each with a 0.2 mm base diameter and a height of 0.7 mm. The numerical calculation of light propagation through the microneedle was performed using the Monte Carlo (MC) simulation, as shown in Figure 1f. The distribution of the normalized light intensity suggested that the microneedle redistributed the LED light along the direction of the needle and delivered it to the surroundings via the tip and side illumination.

The cross‐sectional layer structure of a microneedle in Figure 1g shows a µLED integrated within an underside pocket of the ITO‐coated PLA MNA. The top surface of the MNA was encapsulated by polydimethylsiloxane (PDMS), leaving only the needles exposed for the localized injection of stimulation currents. The stimulation currents and LED power were supplied externally via an FPCB ribbon cable, as shown in Figures S1 and S2 (Supporting Information). The optical transmittance of each layer that the light from the 850 nm µLED passed through before reaching the cortex was measured, as shown in Figure 1h, using the setup in Figure S3 (Supporting Information), along with the cumulative transmittance from µLED to cortex. With at least 86% transmittance in each layer, it is estimated that 60% of the initial optical energy is delivered to the cortex. Despite slight variations in skull thickness, the optical transmittance did not exhibit significant differences among mice aged 6–43 weeks (Figure S4, Supporting Information). Figure 1i displays the emission spectrum of the 840 nm µLED (with its *I*–*V* curve in Figure S5, Supporting Information), where its output irradiance is controlled by varying the diode current from 0 to 30 mA (Figure 1j,k). Supplying a current of 20 mA produced an optical power of 117 mW cm^−2^ from the µLED, 89 mW cm^−2^ from the MNA, and 69 mW cm^−2^ reaching the cortex. This range of irradiance has been shown to be effective for PBM.^[^ ^14b^ ^]^ The optical characteristics measured from multiple MNA devices (#1–4 in Figure 1j) demonstrate consistent optical stimulation intensity across different devices.

### 2.2 Materials and Design of the Hybrid Electro‐optical Stimulator for Neuromodulation

**Figure 2:**
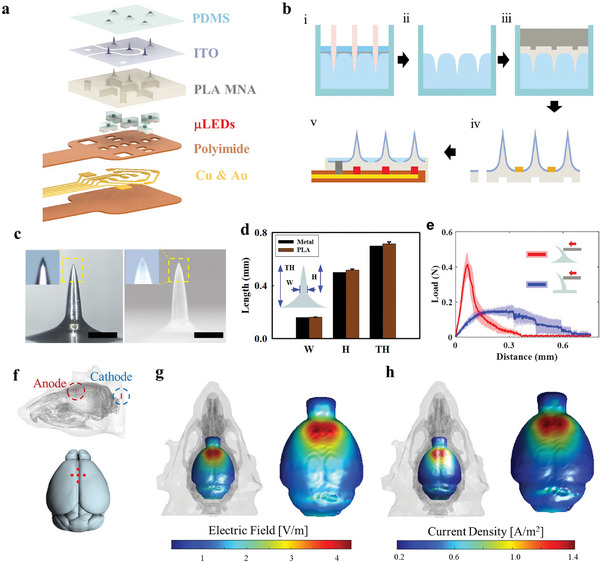
Fabrication and electromechanical characterization of the hybrid electro‐optical stimulator. a) Exploded structure of the stimulator. b) Simplified fabrication process of PLA MNA. Master mold of acupuncture needles (i) is double‐casted (ii) by PLA to form MNA with underside µLED pockets (iii), followed by ITO coating (iv) and assembly with µLED‐mounted FPCB cable and PDMS insulation (v). c,d) Comparison of master microneedle and PLA microneedle, with quantitative analysis of their geometric parameters. Scale bar: 0.2 mm. e) Lateral bending force measured from PLA MNA with (red) and without meniscus‐shaped base (blue). f) Three‐dimensional mouse brain model with skull, scalp, and electrode placement for simulation of electrical stimulation from the device. Simulated distribution of g) electric field and h) current density at the cortex surface. Abbreviations: height (H), total height (TH), width (W), light‐emitting diode (LED), microneedle array (MNA), polydimethylsiloxane (PDMS), polylactide (PLA), and indium tin oxide (ITO).

The exploded structure of the hybrid electro‐optical stimulator is shown in **Figure** **2**a, comprising a PDMS insulation layer, conductive ITO layer, PLA MNA, µLEDs, and a polyimide FPCB with copper and gold interconnections. The fabrication process is illustrated in Figure 2b. A master microneedle fixture, made from 0.2‐mm‐diameter acupuncture needles, was replicated via PDMS double‐casting to create an array of biocompatible and transparent PLA microneedles with backside pockets for housing µLEDs. The bottoms of the microneedles gradually widened to form a meniscus‐like base created by the capillary force and surface tension of the PDMS resin coated over the fixing plate. The microneedles, coated with a biocompatible and conductive ITO layer (200 nm thickness), were then assembled with an µLED‐mounted FPCB cable using optical epoxy before filling the vias to interconnect the FPCB and ITO layers with conductive epoxy (see the Experimental Section and Figures S6–S8, Supporting Information, for more details).

The replication fidelity was evaluated by comparing the shape of the PLA microneedles with that of the original metal microneedles, as shown in Figure 2c. The sharp tip and meniscus‐shaped base were faithfully reproduced by the PLA needles, which was confirmed through the quantitative analysis of geometric parameters, including needle height (*H*), total height (*TH*), and width (*W*), as plotted in Figure 2d. The meniscus‐shaped base of the microneedle significantly enhanced its tolerance against the lateral bending force encountered during handling and surgery compared with the straight needle, as shown in Figure 2e. The maximum bending load, at which irreversible damage occurs to the microneedles, increased from 0.19 to 0.4 N by using the meniscus structure at the base of microneedles.

Numerical analysis was conducted in a 3D mouse model to compute the distribution of cortical activation induced by tDCS on the scalp using MNA (Figure 2f–h). ES (Figure 2g) from the MNA was predicted to elicit a peak current density of 4.2 V m^−1^, and an electric field of 1.5 A m^−2^ on the cortical surface (Figure 2h). The estimated electric field is higher than the threshold intensity required to affect the brain activity as ≈2 V m^−1^,^[^ ^15^ ^]^ while the peak current density is lower than the values known to induce brain injury occurring at 6.3–13 A m^−2^.^[^ ^16^ ^]^ The charge‐injection characteristics of the ITO‐coated MNA were demonstrated by the voltage transient during pulsing in the phosphate‐buffered saline (PBS) solution, as shown in Figure S8 (Supporting Information).

### 2.3 Effects of Hybrid Electro‐optical Stimulation on Ischemic Brain Damage and Functional Recovery Following Stroke in Mice

**Figure 3:**
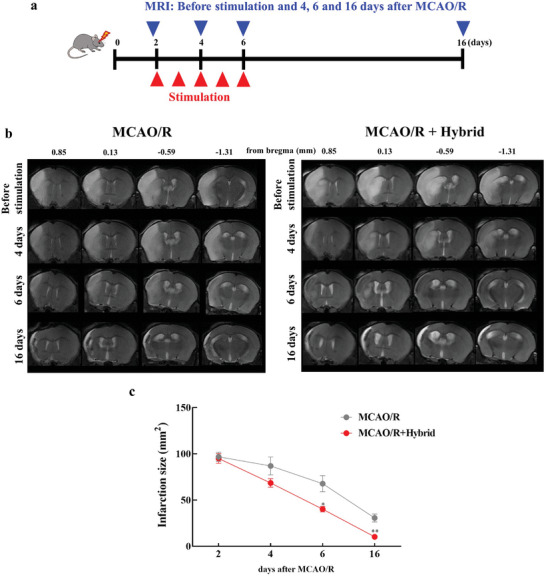
Effects of hybrid electro‐optical stimulation on infarction size following MCAO/R over time. a) Schematic timeline showing the experimental setup and stimulation periods for MCAO/R and hybrid electro‐optical stimulation. MRI scans were performed before stimulation (on day 2) and on days 4, 6, and 16 after MCAO/R. b) Representative T2‐weighted MRI images showing infarct areas in stimulated (hybrid) and non‐stimulated (MCAO/R) groups. c) Quantification of infarction size (mm^2^) over time, demonstrating a significant reduction in infarct volume in the hybrid stimulation group compared to that in the MCAO/R group, particularly on days 4 and 6. All data are represented as mean ± SEM (*n* = 4 per group). Statistical significance was determined using unpaired, two‐tailed Student's *t*‐tests. *P < 0.05, **P < 0.01, versus the MCAO/R group. Abbreviations: middle cerebral artery occlusion/reperfusion (MCAO/R).

**Figure 4:**
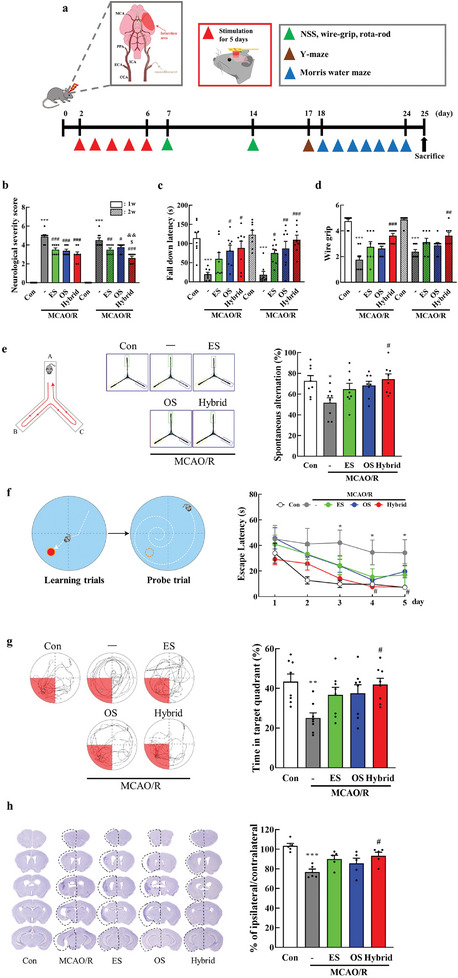
Hybrid electro‐optical stimulation improves functional recovery and brain atrophy after ischemic injury. a) Experimental design. The mice underwent behavior tests to evaluate the recovery of b) neurological deficits and motor function using c) rotarod and d) wire grip tests at 7 and 14 d after ischemic injury (*N* = 8 per group). e) (Left panel) The mice freely explored the Y‐maze for 8 min while being recorded for (middle panel) tracking movement. e) (Right panel) Quantification of spontaneous alternation rate (*N* = 8 per group). f) (Left panel) Spatial learning and memory were evaluated using Morris water maze test. (Right panel) During learning trials, the mice were placed in a water‐filled pool with a hidden platform for learning trial tests and measured escape latency. On probe trial day, mice were conducted to evaluate the time spent in the target quadrant without the platform for 90 s. g (Left panel) During this trial, the mice were recorded for tracking movement. (Right panel) Quantification of the percentage of spent time in the target quadrant (*N* = 8 per group). h) Twenty‐five days after MCAO/R, the obtained brain slices were stained with 0.1% cresyl violet, and brain atrophy was quantified (*N* = 5 per group). All data are represented as mean ± SEM. Statistical significance was determined using one‐way ANOVA with Tukey's post‐hoc test. *P < 0.05 and ***P < 0.001, versus the control group. #*P* < 0.05, ##*P* < 0.01, and ###*P* < 0.001, versus the MCAO/R group. $*P* < 0.05 versus the ES group. &&*P* < 0.01 versus the OS group. Abbreviations: neurological severity score (NSS), control (Con), middle cerebral artery occlusion/reperfusion (MCAO/R), electrical stimulation (ES), and optical stimulation (OS).

The hybrid electro‐optical stimulator was applied to a mouse model of stroke to investigate its potential as a therapeutic intervention against ischemic stroke. Initially, two OS wavelengths were assessed in a mouse model of photothrombotic cortical ischemia to identify the most effective hybrid stimulation for stroke treatment. The results showed that electro‐850 nm hybrid stimulation led to superior improvement in motor functions than electro‐630 nm hybrid stimulation, indicating its suitability for stroke therapy (Figure S10, Supporting Information). Subsequently, we examined the effects of the hybrid electro‐850 nm optical stimulation on neurological, motor, and cognitive impairments after middle cerebral artery occlusion and reperfusion (MCAO/R) injury. The mice underwent daily sessions of hybrid electro‐optical stimulation, which included electrical (83.33 µA mm^−2^, charge density: 50 kC m^−2^) and optical stimulations (89 mW cm^−2^, 850 nm) on the hairless scalp for 10 min over 5 d, starting 48 h after stroke induction. To determine the comparability of the initial lesion size and to monitor lesion progression throughout the treatment period, we quantified the infarction size in both the MCAO/R and hybrid stimulation groups. T2‐weighted magnetic resonance imaging (MRI) scans were conducted before stimulation (on day 2) and on days 4, 6, and 16 after MCAO/R to evaluate infarction areas before and during hybrid electro‐optical stimulation (**Figure** **3**a). MRI images revealed comparable infarction areas in both the MCAO/R and hybrid stimulation groups at 2 d post‐MCAO/R, prior to stimulation (Figure 3b). Over the course of the experiment, infarction sizes decreased in both groups. However, at later time points (days 4, 6, and 16), the hybrid stimulation group exhibited a more pronounced reduction in infarction size compared to the nonstimulated MCAO/R group. Quantification of infarction size demonstrated significantly smaller lesion volumes in the hybrid group than in the MCAO/R group both 6 d (*P < 0.05, [*P* = 0.0204]) and 16 d (**P < 0.01, *P* = 0.0048) after MCAO/R (Figure 3c). These results indicate that hybrid electro‐optical stimulation effectively reduces ischemic brain damage, supporting its therapeutic potential for stroke recovery. Neurological and motor outcomes were evaluated at 7 and 14 d poststroke (**Figure** **4**a). Notably, as illustrated in Figure 3b, the neurological severity score (NSS) demonstrated significant improvement with ES alone, OS alone, and hybrid stimulation at both time points. Particularly noteworthy was the substantial enhancement in neurological damage observed with hybrid stimulation compared with electrical and optical stimulation alone at two weeks post‐ischemic brain injury (Figure 4b). Furthermore, hybrid stimulation significantly augmented the impaired motor function at 7 and 14 d (Figure 4c,d). Collectively, these findings indicate that the hybrid electro‐optical stimulation severely attenuated the neurological and motor function deficits induced by focal cerebral ischemia, which increased over time.

Subsequently, we evaluated the cognitive function 17 d poststroke using the Y‐maze and Morris water maze tests (Figure 4e–g). The Y‐maze test, designed to evaluate spatial working memory, involved mice freely navigating a Y‐shaped platform, and their entries into each arm were recorded for 8 min, as shown in Figure 4e. The stroke mice exhibited significantly reduced spontaneous alternation entry into each arm compared to control groups and this phenotype was notably reversed by hybrid stimulation (51.57 ± 1.62% vs 74.45 ± 1.66% in the stroke and hybrid stimulation groups, respectively, *P* < 0.05 [*P* = 0.02], Figure 4e). In addition, no other significant differences in the total number of arm entries were observed between the groups (Figure S11, Supporting Information). Furthermore, we investigated whether hybrid stimulation could ameliorate spatial learning and memory deficits in a stroke model using the Morris water maze test (Figure 4f,g). In the learning trial, the stroke mice took longer to find the submerged platform than the control groups (*P* < 0.05 [*P* = 0.019]), whereas hybrid‐stimulated stroke mice demonstrated a shorter latency to locate the hidden platform (*P* < 0.05 [*P* = 0.01]). During the probe trial on the following day, the stroke mice spent less time in the target quadrant (*P* < 0.01 [*P* = 0.008]) after platform removal. Conversely, hybrid‐stimulated stroke mice exhibited a significantly increased time spent in the target quadrant (*P* < 0.05 [*P* = 0.017]) compared to the MCAO/R mice. These findings suggest that hybrid stimulation during the acute and subacute phases of stroke improved spatial learning and memory during the chronic phase.

We further examined whether improved brain function in stroke mice following hybrid stimulation was accompanied by histological recovery. The changes in brain morphology or volume 25 d poststroke were assessed using Nissl staining (Figure 4h). The MCAO/R mice exhibited significantly lower brain volume than control mice (*P* < 0.001), while hybrid‐stimulated stroke mice displayed a notable restoration of brain volume compared to MCAO/R‐only mice (*P* < 0.05 [*P* = 0.044]), indicating the potential neuroprotective effects of hybrid stimulation.

Overall, these findings indicate the therapeutic potential of hybrid electro‐optical stimulation in enhancing neurological, motor, and cognitive functions, as well as reducing brain atrophy following ischemic stroke.

### 2.4 Neuroprotective Effects of Hybrid Electro‐optical Stimulation on Ischemic Brain Damage

**Figure 5:**
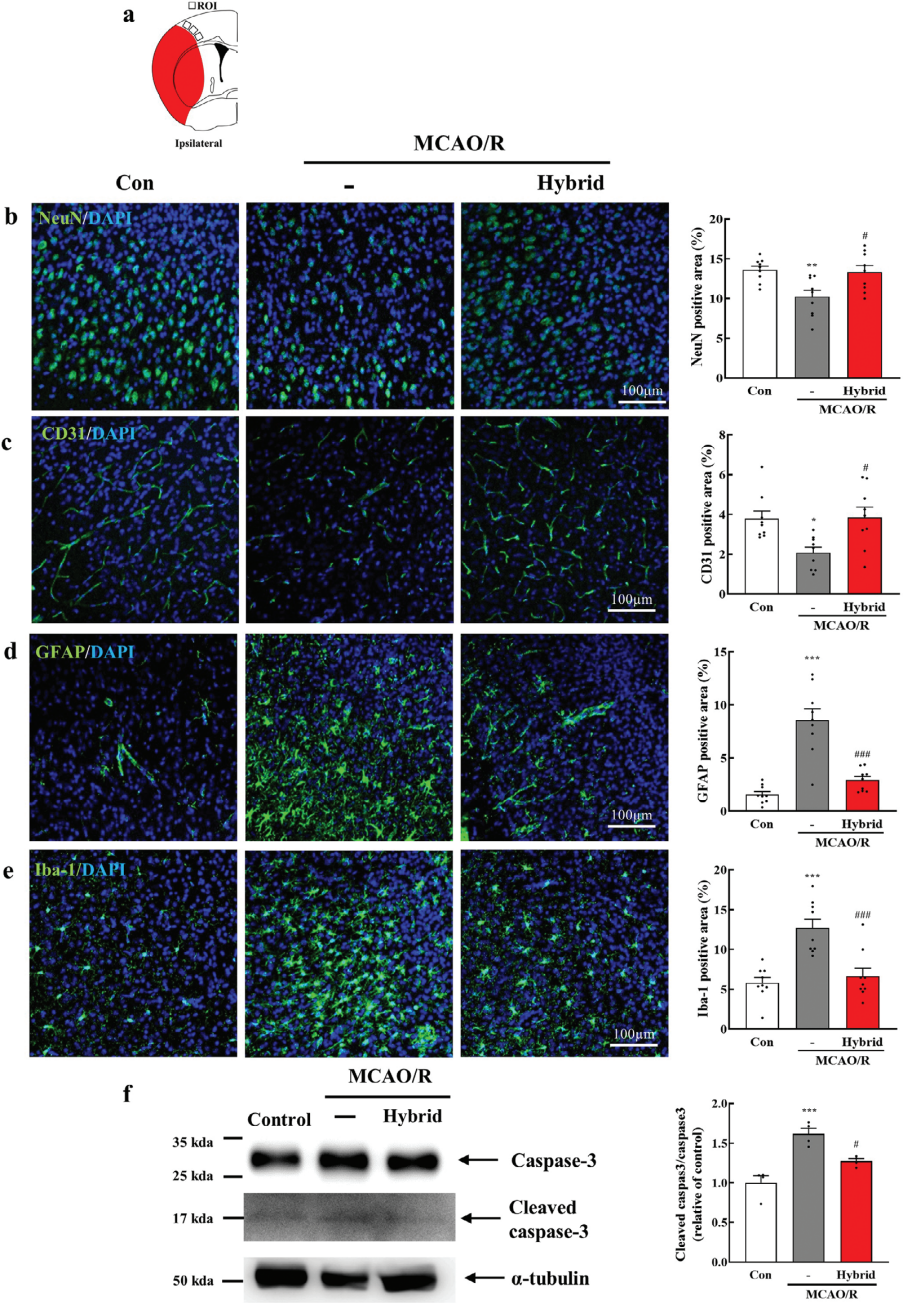
Hybrid electro‐optical stimulation enhances neuroprotective effects on the ischemic brain. a) Illustration of the ROI for observing immunostained markers in the peri‐infarct regions 7 d after MCAO/R. Representative images and quantification of b) NeuN (Neuronal cell), c) CD31 (endothelial cell), d) GFAP (astrocyte), and e) Iba‐1 (microglia). *N* = 9 images for each group. f) Representative western blot images showing caspase‐3 (30 kDa), cleaved caspase‐3 (17 kDa), and α‐tubulin (50 kDa, loading control), as well as the quantification of caspase‐3 and cleaved caspase‐3 expression relative to α‐tubulin (*N* = 4 for each group). All data are represented as mean ± SEM. Statistical significance was determined using one‐way ANOVA with Tukey's post‐hoc test. *P < 0.05, **P < 0.01, and ***P < 0.001, versus the control group. #*P* < 0.05, ##*P* < 0.01, and ###*P* < 0.001, versus the MCAO/R group. Abbreviations: control (Con), middle cerebral artery occlusion/reperfusion (MCAO/R), region of interest (ROI), neuronal cell (NeuN), cluster differentiation 31 (CD31), glial fibrillary acidic protein (GFAP), ionized calcium‐binding adapter molecule‐1 (Iba‐1), and 4′,6‐diamidino‐2‐phenylindole (DAPI).

To investigate the neuroprotective potential of hybrid stimulation, we analyzed the brain cells in the ischemic cortex by measuring the expression levels of various immunostained markers in the peri‐infarct regions one day after hybrid stimulation (day 7 poststroke). These markers included NeuN (neuronal cells), CD31 (endothelial cells), GFAP (astrocytes), and Iba‐1 (microglia). Fluorescence microscopy results revealed that hybrid stimulation significantly increased the number of neuronal cells (*P* < 0.05 [*P* = 0.016]) and CD31‐positive cells (*P* < 0.05 [*P* = 0.013]) compared to those in the MCAO/R mice (**Figure** **5**b,c). Furthermore, we observed significantly decreased GFAP‐positive (*P* < 0.001) and Iba‐1‐positive (*P* < 0.001) areas in the peri‐infarct cortex poststroke in the hybrid‐stimulated group compared to those in the MCAO/R mice (Figure 5d,e). These results suggest that hybrid stimulation has the potential to attenuate glial cell activation while preserving neurons and blood vessels in the ischemic brain. To further confirm the effect of hybrid electro‐optical stimulation on apoptosis, we evaluated cleaved caspase‐3 and caspase‐3 expression levels. Western blot analysis revealed a significant increase in the cleaved caspase‐3/caspase‐3 ratio (*P* < 0.001) in the MCAO/R group compared to that in the control group. However, hybrid stimulation significantly reduced the cleaved caspase‐3/caspase‐3 ratio (*P* < 0.05 [*P* = 0.017]), indicating that hybrid stimulation effectively attenuates apoptosis in the ischemic brain (Figure 5f).

To investigate the molecular mechanisms underlying these biological changes, we performed RNA sequencing (RNA‐seq) on the peri‐infarct regions from the control, MCAO/R, and hybrid‐stimulated groups. Differential gene expression analysis identified 667 significantly altered genes in the hybrid‐stimulated group compared to MCAO/R mice, with 171 upregulated and 496 downregulated genes (Figure S12a,b, Supporting Information). Gene Ontology (GO) enrichment analysis revealed that upregulated genes were associated with neuronal growth and repair processes, such as neuron projection development (GO: 0031175), axon guidance (GO: 0007411), synaptic plasticity (GO: 0048168), and axon extension (GO: 0048675) (Figure S12c, Supporting Information). Similarly, Kyoto Encyclopedia of Genes and Genomes (KEGG) pathway analysis highlighted enrichment in neuroplasticity‐related pathways, including ErbB signaling (mmu04012), neurotrophin signaling (mmu04722), and axon guidance (mmu04360), which are crucial for neuronal regeneration and functional recovery (Figure S12d, Supporting Information). Consistent with transcriptomic findings, immunofluorescence staining demonstrated increased neurogenesis and proliferation in hybrid‐stimulated mice. Specifically, hybrid stimulation significantly enhanced the co‐localization of Ki67 (a marker of proliferation) and NeuN (a neuronal marker), as well as DCX (a marker of neuronal precursors) and NeuN, in the peri‐infarct cortex and dentate gyrus (Figure S13, Supporting Information). These results suggest that hybrid stimulation actively promotes the generation of new neurons and supports their survival, contributing to recovery after ischemic injury. Conversely, downregulated genes were linked to apoptotic processes (GO: 0006915), T cell‐mediated cytotoxicity (GO: 0001913), and innate immune responses (GO: 0045087), suggesting a reduction in cell death and inflammation (Figure S12e, Supporting Information). KEGG pathway analysis also revealed suppression of immune‐related pathways, such as PI3K‐Akt signaling (mmu04151) and MicroRNAs in cancer (mmu05206), which are associated with stress responses and immune activation (Figure S12f, Supporting Information). These molecular changes indicate that hybrid stimulation mitigates harmful immune responses while fostering a neuroprotective environment conducive to recovery.

### 2.5 Enhancing Effects of Hybrid Electro‐optical Stimulation on the Glymphatic Function in Ischemic Brain Injury

**Figure 6:**
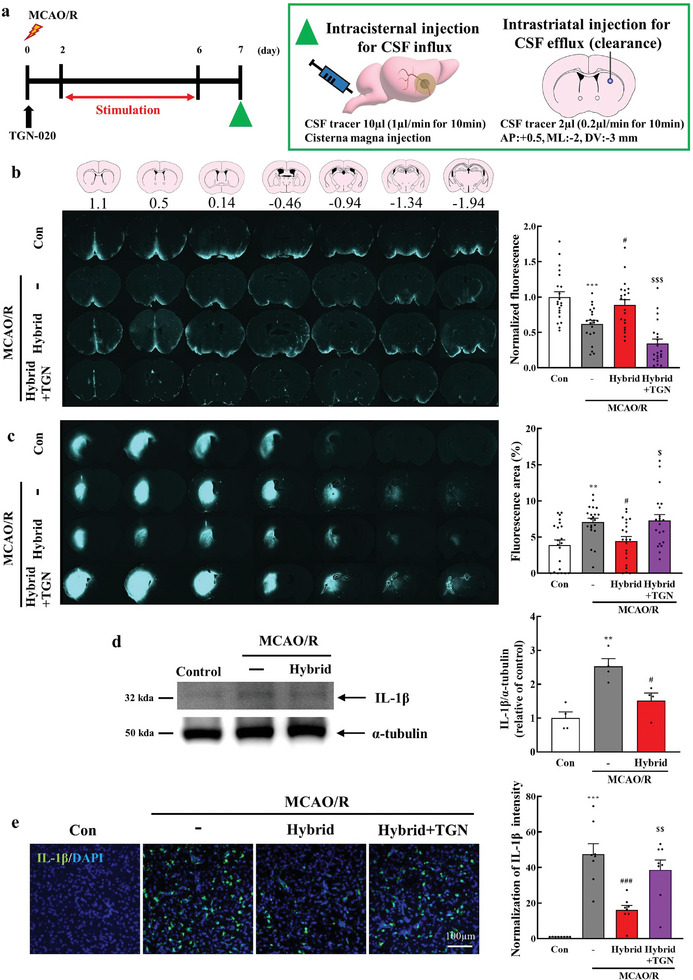
Hybrid electro‐optical stimulation restores glymphatic function in the ischemic brain. a) (Left panel) Experimental design. Mice were intraperitoneally administered TGN‐020 (200 mg kg^−1^) 10 min after MCAO/R for inhibition of AQP4. (Right panel) The illustration shows that a fluorescence tracer was injected into the cisterna magna or brain parenchyma to assess glymphatic function 7 d after MCAO/R. Seven coronal brain sections at 1.1, 0.5, 0.14, −0.46, −0.94, −1.34, and −1.94 from bregma per animal were quantified. b) Representative images show the fluorescence tracer influx and average quantification of fluorescence intensity of CSF tracer efflux into the brain parenchyma (*N* = 21 brain slices per group). c) Representative images indicate the fluorescence tracer of CSF clearance and quantification of percentage area of the accumulated CSF tracer per brain slice (*N* = 21 brain slices per group). d) Representative western blot images showing IL‐1β (32 kDa) and α‐tubulin (50 kDa, loading control), along with the quantification of IL‐1β expression relative to α‐tubulin (*N* = 4 for each group). e) Representative images show proinflammatory cytokine, IL‐1β staining in the peri‐infarct area, and quantification of fluorescence intensity (*N* = 8 images for each group). All data are represented as mean ± SEM. Statistical significance was determined using one‐way ANOVA with Tukey's post‐hoc test. **P < 0.01 and ***P < 0.001 versus the control group. #*P* < 0.05 and ###*P* < 0.001, versus the MCAO/R group. $*P* < 0.05, $$*P* < 0.01, and $$$*P* < 0.001, versus the hybrid group. Abbreviations: cerebrospinal fluid (CSF), control (Con), middle cerebral artery occlusion/reperfusion (MCAO/R), TGN‐020 (TGN), Interleukin‐1 beta (IL‐1β), and 4′,6‐diamidino‐2‐phenylindole (DAPI).

To understand the advantageous effects of hybrid electro‐optical stimulation on ischemic brain injury, we examined the alterations in glymphatic function induced by hybrid stimulation. On the day following hybrid stimulation (day 7 poststroke), the mice received injections of a CSF fluorescence tracer into the cisterna magna and brain parenchyma to assess the CSF influx and ISF clearance (**Figure** **6**a). Thirty minutes after the infusion of the CSF tracer, whole‐brain sections were collected. The penetration of the CSF tracer into the brain parenchyma was significantly diminished in MCAO/R mice compared to the control group (*P* < 0.001). The hybrid stimulation notably enhanced the tracer inflow compared to MCAO/R mice (*P* < 0.05 [*P* = 0.028]), and this effect was significantly counteracted in mice treated with hybrid stimulation + TGN‐020, a known AQP4 inhibitor (Figure 6b, *P* < 0.001). Subsequently, we assessed whether hybrid stimulation affected the clearance rate of solutes in the striata of stroke mice. We also administered an intrastriatal injection to evaluate ISF clearance after MCAO/R, following the same timeline as in the CSF influx experiment described above (Figure 6a). After 2 h of cycling and sample processing, representative sections were analyzed to evaluate the tracer residues within the brain parenchyma comprehensively. The residual tracer in the brain was significantly elevated in MCAO/R mice compared to the control mice (*P* < 0.01 [*P* = 0.007]), which was significantly reversed by hybrid stimulation (*P* < 0.05 [*P* = 0.035]). Furthermore, mice treated with hybrid stimulation + TGN‐020 exhibited significantly reduced tracer drainage compared to those treated with hybrid stimulation alone. (Figure 6c; *P* < 0.05 [*P* = 0.018]). Correspondingly, western blot experiments demonstrated that the upregulation of the inflammatory cytokine IL‐1β following MCAO/R was reduced in hybrid‐stimulated mice (Figure 6d). Immunofluorescence results showed the same trend, and this effect was significantly reversed by TGN‐020 treatment (Figure 6e, *P* < 0.01 [*P* = 0.005]). These findings suggest that hybrid electro‐optical stimulation recovers impaired glymphatic function following MCAO/R, potentially leading to reduced levels of proinflammatory cytokines.

**Figure 7:**
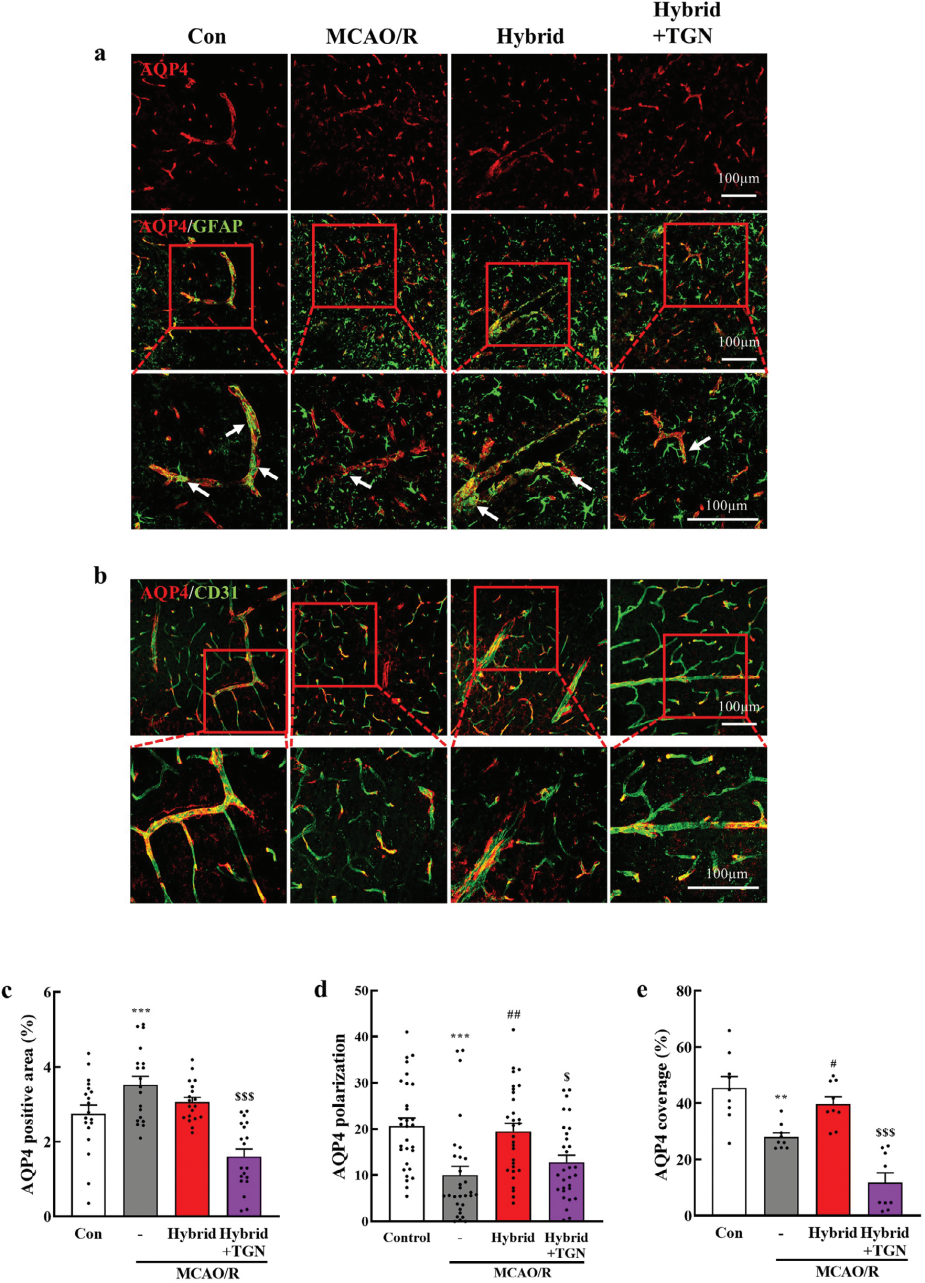
Hybrid electro‐optical stimulation recovers AQP4 polarization in the ischemic brain. a) Representative images of AQP4/GFAP immuno‐staining in the peri‐infarct cortex 7 d after MCAO/R. White arrows of 40× magnification images represent the localized AQP4 on astrocytic endfeet. b) Representative images show AQP4/CD31 in the peri‐infarct cortex 7 d after MCAO/R and 40× magnification images represent AQP4 coverage of vessels. c–e) Quantification of the AQP4‐positive area (*N* = 19 images per group), perivascular AQP4 polarization (*N* = 30 vessels per group), and AQP4 coverage of CD31 (*N* = 9 images per group). All data are represented as mean ± SEM. Statistical significance was determined using one‐way ANOVA with Tukey's post‐hoc test. **P < 0.01 and ***P < 0.001 versus the control group. #*P* < 0.05 and ##*P* < 0.01, versus the MCAO/R group. $*P* < 0.05 and $$$*P* < 0.001, versus the Hybrid group. Abbreviations: control (Con), middle cerebral artery occlusion/reperfusion (MCAO/R), TGN‐020 (TGN), Aquaporin‐4 (AQP4), glial fibrillary acidic protein (GFAP), and cluster differentiation 31 (CD31).

The typical localization of the water channel AQP4 is on the endfeet of perivascular astrocytes; however, the glymphatic system can be impaired due to mislocalization of AQP4.^[^ ^10^ ^]^ Consequently, to investigate the impact of hybrid stimulation on the glymphatic system function, we examined the levels of AQP4 protein and AQP4 polarization in the peri‐infarct regions one day after hybrid stimulation (7 d poststroke). AQP4 expression was significantly higher in MCAO/R mice than in control mice (Figure S14, Supporting Information, *P* < 0.01), but no differences were observed among the MCAO/R, ES, OS, and hybrid stimulation groups (Figure S14, Supporting Information). We then analyzed perivascular AQP4 polarization using confocal images of GFAP and AQP4 (Figure S15, Supporting Information), providing a relative assessment of AQP4 localization on perivascular astrocyte endfeet. Double staining for AQP4 and GFAP revealed significantly reduced AQP4 polarization in MCAO/R mice compared to that in the control group (Figure S14, Supporting Information, *P* < 0.001). The hybrid‐stimulated group exhibited increased AQP4 polarization compared to the MCAO/R group (*P* < 0.001), ES (*P* < 0.001), and OS treatments (*P* < 0.01 [*P* = 0.004]), suggesting a synergistic effect of electrical and optical stimulation on AQP4 polarization. This increased AQP4 polarization effect was reversed by pharmacological inhibition of AQP4 (**Figure** **7**, *P* < 0.05 [*P* = 0.038]). Subsequently, we quantified AQP4 intensity in the perivascular area (AQP4 coverage), revealing that hybrid stimulation notably enhanced AQP4 coverage of CD31 (a vascular endothelial cell marker) compared to that in the MCAO/R group (Figure 7b,e, *P* < 0.05 [*P* = 0.045]). However, this effect was significantly reversed by TGN‐020 treatment (*P* < 0.001). These results suggest that hybrid stimulation restores impaired glymphatic function after MCAO/R, not by altering AQP4 expression, but by improving its localization to astrocyte endfeet.

**Figure 8:**
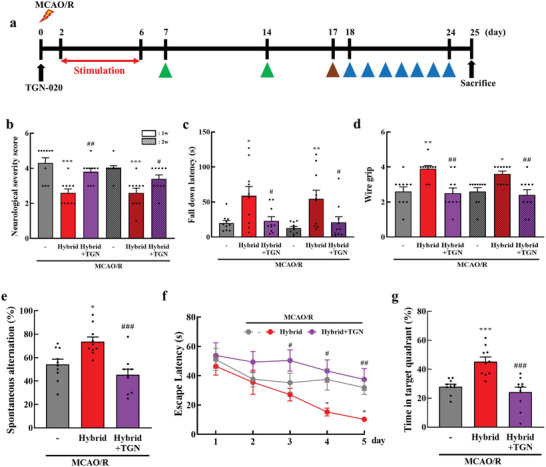
AQP4 inhibition reverses the beneficial effects of hybrid electro‐optical stimulation on ischemic injury. a) Experimental design. Mice were intraperitoneally administered TGN‐020 (200 mg kg^−1^) 10 min after MCAO/R and treated with hybrid electro‐optical stimulation for 5 d. The mice underwent behavior tests to evaluate the recovery of b) neurological deficits and motor function using c) rotarod and d) wire grip tests at 7 and 14 d after ischemic injury. e) Mice freely explored the Y‐maze for 8 min and quantified the spontaneous alternation rate. Spatial learning and memory were evaluated using the Morris water maze test. f) Mice were placed in a water‐filled pool with a hidden platform for learning trial tests and measured escape latency during learning trials. g) On day 7, a probe trial test was conducted to evaluate the time the mice spent in the target quadrant without the platform for 90 s. All data are represented as mean ± SEM. *N* = 10 per group. Statistical significance was determined using one‐way ANOVA with Tukey's post‐hoc test. *P < 0.05, **P < 0.01, and ***P < 0.001, versus the MCAO/R group. #*P* < 0.05, #*P* < 0.05, and ###*P* < 0.001, versus the MCAO/R group. Abbreviations: middle cerebral artery occlusion/reperfusion (MCAO/R), and TGN‐020 (TGN).

Hybrid stimulation appears to increase AQP4 polarization, enhance glymphatic function, and ultimately reduce poststroke brain damage. To validate this hypothesis, we investigated whether TGN‐020 could reverse the beneficial effects of hybrid stimulation following ischemic brain injury (**Figure** **8**a). The administration of TGN‐020 (200 mg kg^−1^, i.p.) reversed the neurological and motor deficits induced by hybrid stimulation 7 and 14 d after MCAO/R (Figure 8b–d). In the Y‐maze test, the increase in spontaneous alternation by hybrid stimulation was significantly decreased by TGN‐020 treatment (Figure 8e, *P* < 0.001). In the learning trial of Morris water maze test, hybrid‐stimulated stroke mice showed a significant decrease in escape latency over 5 d compared to MCAO/R mice (Figure 8f, *P* < 0.05 [*P* = 0.016]), whereas mice treated with hybrid stimulation + TGN‐020 demonstrated a longer latency to locate the hidden platform (*P* < 0.01 [*P* = 0.002]). The next day, mice treated with hybrid stimulation + TGN‐020 spent significantly less time in the target quadrant than mice in the hybrid treatment group (Figure 8g, *P* < 0.001). These results suggest that AQP4 and the glymphatic system play a critical role in poststroke recovery. By improving AQP4 polarization and glymphatic function, hybrid electro‐optical stimulation enhances the clearance of harmful substances and reduces brain inflammation. This improved clearance likely contributed to the observed improvements in neurological, motor, and cognitive functions. The reversal of these benefits by TGN‐020 further underscores the importance of glymphatic function in mediating the therapeutic effects of hybrid stimulation.

## 3 Discussion

In this study, we developed a device that allows simultaneous electrical and optical stimulations. The hybrid electro‐optical stimulator for stroke rehabilitation features an advanced design that delivers targeted stimulation and boosts glymphatic function and brain recovery processes. Using a sophisticated microneedle array, this system precisely controls both light and ESs, thereby directly influencing tissue and functional recovery. Moreover, the stimulator critically modulates AQP4 polarization, which is essential for enhancing glymphatic clearance, thus improving brain recovery from ischemic damage and potentially reducing neurological, motor, and cognitive deficits. This dual‐modality approach not only lessens the impact of ischemic injury, but also supports enhanced recovery and redefines therapeutic interventions for brain injuries.

The development of a hybrid electro‐optical stimulator featuring a transparent and conductive MNA enables noninvasive hybrid stimulation of the cortical area for poststroke rehabilitation. Electrical stimulation delivered through microneedles has been reported to provide focused cortical activation compared with traditional planar electrodes.^[^ ^17^ ^]^ Moreover, each needle was microstructured with a µLED pocket on its underside to ensure precise spatial alignment between the microneedles and µLEDs. This design allows for the simultaneous delivery of electrical and optical stimulations to the same location, thereby maximizing the synergistic effectiveness of stimulation. Initially, two OS wavelengths were selected to assess the most effective hybrid stimulation for stroke treatment in a mouse model of photothrombotic cortical ischemia. Near‐infrared µLEDs (850 nm) were chosen for this study due to their relatively high optical transmittance through biological tissues, and visible µLEDs (630 nm) were selected because they were found to be effective in improving ischemic brain injury in previous studies.^[^ ^14^ ^]^ The results showed that electro‐850 nm hybrid stimulation led to superior improvement in motor functions compared with electro‐630 nm hybrid stimulation, indicating its suitability for stroke therapy. Therefore, the hybrid electro‐850 nm OS was selected for further research. Through a layer‐by‐layer optical characterization of the actual media present in the device and animal, it was determined that at least 60% of the initial optical energy from the 850 nm µLED reaches the cortex. This ensured a sufficient range of optical energy to modulate cortical functions using moderate levels of LED driving currents.

Previous research has primarily focused on either electrical or optical stimulation individually, showing benefits in stroke recovery;^[^ ^13^ ^]^ however, our results indicate that a combination of these modalities yields a more substantial effect than either stimulation alone. In our study, we placed stimulation needles on the hairless scalp, arranged 2 mm apart in both the medial‐lateral (ML) and anterior‐posterior (AP) directions, with four needles encircling the center needle (ML: 0 mm, AP: +0.5 mm from the bregma). This configuration targets the area of the scalp innervated by the trigeminal nerve and motor cortex, which may facilitate its application in clinical settings in the future. Stimulation points innervated by the trigeminal nerve have been reported to be an effective approach to stroke therapy and can be integrated with other modalities such as transcranial magnetic stimulation (TMS) for enhanced effects.^[^ ^18^ ^]^ Brain stimulation for stroke rehabilitation typically targets the motor cortex.^[^ ^19^ ^]^ In our protocol, mice received daily hybrid electro‐optical stimulation sessions, which included electrical (83.33 µA mm^−2^, charge density: 50 kC m^−2^) and optical stimulations (89 mW cm^−2^, 850 nm) for 10 min across 5 d, beginning 48 h after MCAO/R. Despite modifications in stimulating the scalp instead of the skull and reducing the duration to 10 min daily,^[^ ^13^, ^14^ ^]^ our hybrid electro‐optical stimulation significantly enhanced neurological, motor, and cognitive functions following ischemic stroke in mice. Notably, improvements in neurological and motor functions intensified over time, indicating a sustained therapeutic impact. Furthermore, hybrid stimulation appeared to alleviate brain atrophy, as evidenced by brain volume recovery and exerted neuroprotective effects by modulating various cellular responses in the ischemic cortex. Hybrid stimulation increased endothelial cells and decreased astrocytes, microglia, and apoptotic cells in stroke‐damaged lesions, which is consistent with a previous report that optic stimulation alone alleviates tissue damage and minimizes functional deficits by preserving blood vessels and attenuating glial cell activation after focal cerebral ischemia.^[^ ^14^ ^]^ As previously documented, preserving cerebrovascular blood vessels and reducing neuroinflammation via glial cells are critical strategies for recovery after ischemic stroke.^[^ ^20^ ^]^ Recent research has highlighted the important protective role of microglia in ischemic stroke.^[^ ^43^ ^]^ Our RNA‐seq analysis revealed that hybrid electro‐optical stimulation reduces the activation of pathways related to apoptosis and cell death while simultaneously enhancing pathways associated with neurogenesis. These transcriptomic findings are corroborated by our immunofluorescence results, which demonstrated increased neuronal proliferation and survival in mice treated with hybrid stimulation. Specifically, hybrid stimulation significantly increased co‐localization of Ki67 and NeuN, as well as DCX and NeuN, in the cortex and dentate gyrus. These findings confirm that hybrid stimulation promotes neurogenesis and cell proliferation, complementing the RNA‐seq data that identified the activation of neurogenic pathways. Collectively, these results underscore the therapeutic potential of hybrid stimulation in creating a neuroprotective environment by reducing cell death and enhancing neuronal regeneration. Overall, hybrid stimulation has the potential to attenuate glial cell activation and death, preserve blood vessels in the ischemic brain, and improve tissue and functional outcomes following ischemic stroke.

Previous research has shown that glymphatic system dysfunction contributes to the accumulation of toxic metabolites and proinflammatory cytokines and delayed recovery poststroke.^[^ ^10^ ^]^ Consistent with our findings, previous studies have noted that CSF influx remains sluggish for up to 7 d following ischemic stroke.^[^ ^21^ ^]^ In contrast, a recent study reported a rapid increase in CSF influx into the glymphatic system immediately after an ischemic insult.^[^ ^22^ ^]^ Another study highlighted that the glymphatic system could be compromised in areas of secondary damage at 1 and 7 d poststroke.^[^ ^21^ ^]^ Further observations showed severe impairment of perivascular influx 2 d after MCAO, which was partially recovered by day 7. Consequently, we applied hybrid stimulation to enhance glymphatic function for 5 d beginning 2 d after MCAO/R, observing significant changes in the glymphatic system and proinflammatory cytokine IL‐1β levels. Several studies have investigated different interventions aimed at enhancing glymphatic clearance, focusing on pharmacological and neuromodulation approaches.^[^ ^23^ ^]^ Xie et al. demonstrated that blocking adrenergic receptors with a cocktail of antagonists enhances glymphatic function in mice.^[^ ^23a^ ^]^ In addition, noninvasive brain stimulation techniques, such as PBM,^[^ ^24^ ^]^ focused ultrasound stimulation,^[^ ^25^ ^]^ and TMS,^[^ ^26^ ^]^ have been shown to improve CSF drainage and cognitive functions in AD models. Our study revealed that hybrid stimulation significantly enhances CSF influx and ISF clearance, and lowers IL‐1β levels, as indicated by the reduced residual tracer in the brain parenchyma of treated mice compared to those that only received MCAO/R. This suggests that hybrid stimulation effectively enhances the bulk flow mechanisms that facilitate the removal of brain metabolites.

AQP4 is a key molecular component of the glymphatic system.^[^ ^8c^ ^]^ In the context of ischemic stroke, the mislocalization of perivascular AQP4 intensifies cerebral edema and disrupts glymphatic function, leading to an accumulation of amyloid‐β and proinflammatory cytokines.^[^ ^27^ ^]^ Functionality of the glymphatic system depends heavily on AQP4 polarization. Our findings indicate that hybrid electro‐optical stimulation can effectively modulate the glymphatic system and AQP4 polarization, which is reversed by the AQP4 inhibitor TGN‐020. Interestingly, despite increased AQP4 polarization due to the hybrid treatment, there was no change in AQP4 expression levels, suggesting that hybrid electro‐optical stimulation may specifically influence AQP4 polarization without altering its overall expression. Moreover, improvements in neurological, motor, and long‐term outcomes after stroke via hybrid stimulation were significantly reversed by AQP4 inhibition, indicating that recovery from functional brain damage after stroke through hybrid stimulation is linked to AQP4. However, AQP4 plays a complex dual role in the pathology of ischemic stroke. Various studies, including those on AQP4 inhibition using inhibitors or knockout (KO) models, have shown reduced brain edema and ischemic brain injury.^[^ ^28^ ^]^ Manley et al. observed that brain edema diminished in AQP4 KO mice 24 h after permanent MCAO.^[^ ^29^ ^]^ Furthermore, TGN‐020 alleviated glymphatic dysfunction by inhibiting astrocyte proliferation and AQP4 polarity at 48 h post‐MCAO/R.^[^ ^28^ ^]^ Conversely, other studies have reported an adverse role of AQP4 in ischemic stroke pathology. AQP4 KO mice exhibited significant astrocyte swelling, increased infarct size, and severe CA1 neuronal loss, exacerbating brain damage 72 h post‐MCAO/R.^[^ ^30^ ^]^ In addition, AQP4‐deficiency mice showed greater neutrophil infiltration and microglial activation than wild‐type mice at 24 and 72 h post‐MCAO/R.^[^ ^31^ ^]^ The dual role of AQP4 in stroke progression is critical to understanding the variable effects of TGN‐020 in ischemia models. While previous studies have shown that early intervention with TGN‐020 in ischemia models can reduce brain edema and improve infarct volume,^[^ ^28^ ^]^ our results demonstrate that TGN‐020 administration reverses the beneficial effects of hybrid stimulation during the poststroke recovery phase. This apparent contradiction can be explained by the dual and time‐dependent role of AQP4 in stroke pathophysiology. In the acute phase of ischemic stroke, AQP4 facilitates water influx into brain tissue, exacerbating edema. Thus, early inhibition of AQP4 with TGN‐020 can be beneficial by limiting this initial edema formation.^[^ ^28^ ^]^ However, our study focuses on the subacute to chronic phases of stroke recovery, where AQP4 function transitions to supporting glymphatic clearance of metabolic waste and excess fluid. During this later phase, our hybrid electro‐optical stimulation appears to enhance AQP4 polarization, promoting efficient glymphatic function without altering overall AQP4 expression. By inhibiting AQP4 at this stage, TGN‐020 disrupts the improved glymphatic clearance induced by our hybrid stimulation, thereby reversing its beneficial effects on neurological, motor, and long‐term outcomes. These findings highlight the complex, time‐dependent role of AQP4 in stroke pathophysiology and recovery. They suggest that while AQP4 inhibition may be beneficial in the acute phase, maintaining or enhancing AQP4 function during the recovery phase is crucial for optimal outcomes. This temporal dynamic underscores the importance of considering the timing of interventions in stroke treatment strategies and provides insight into the mechanisms through which hybrid stimulation promotes recovery by enhancing glymphatic function.

Our study emphasizes the crucial role of AQP4 polarization in glymphatic function and its modulation by hybrid stimulation. While TGN‐020 has been widely employed in glymphatic system research, it is important to note that its effects are not lymphoid‐specific, as it influences both AQP4 expression and polarization. This dual action introduces some complexity in isolating the specific contributions of the glymphatic system to the observed therapeutic effects. Nonetheless, TGN‐020 remains a valuable pharmacological tool for studying glymphatic function, as demonstrated in various neurological models,^[^ ^40^ ^]^ including ischemic stroke.^[^ ^6^, ^41^ ^]^ In our experiments, TGN‐020 administration reversed the improvements induced by hybrid stimulation in AQP4 polarization, glymphatic clearance, and poststroke recovery outcomes. These results highlight the significant role of the glymphatic system in mediating the therapeutic effects of hybrid stimulation. However, the limitations of TGN‐020 necessitate further studies using more specific approaches, such as AQP4 knockout models or polarization‐targeted tools, to elucidate the precise mechanisms underlying these effects. Despite these challenges, our findings strongly support the hypothesis that hybrid stimulation enhances glymphatic system function, thereby reducing inflammation and facilitating recovery. By advancing the understanding of AQP4's dual role, this study contributes to the growing body of evidence supporting glymphatic system‐targeted therapies in ischemic stroke.

It is important to note that glymphatic function and AQP4 polarization are subject to circadian control.^[^ ^42^ ^]^ While our hybrid stimulation approach may potentially influence these circadian‐controlled processes, it is crucial to emphasize that both the hybrid stimulation and sham‐operated groups received treatment during the same time window. This consistent timing across groups ensures that any observed differences in glymphatic function and AQP4 polarization can be attributed to the effects of hybrid stimulation rather than circadian variations. The significant enhancement of glymphatic function observed in the hybrid stimulation group compared to the sham‐operated group, despite the identical timing of interventions, strongly supports the efficacy of our approach. It is worth noting that our experiments were conducted during the light phase, corresponding to the resting period of nocturnal rodents when glymphatic activity is typically enhanced. Therefore, future studies could explore the efficacy of the hybrid stimulation approach at different time points across the circadian cycle to further optimize treatment protocols and potentially uncover any interactions between the stimulation effects and circadian rhythms.

This study demonstrated promising results in enhancing poststroke glymphatic function and functional outcomes using hybrid electro‐optical stimulation. However, there remain several limitations, necessitating further research. First, it did not fully explain the mechanism by which hybrid electro‐optical stimulation specifically targets and modulates the polarization of AQP4. AQP4 polarization is essential for proper glymphatic function; however, the precise biophysical and molecular pathways by which electrical and optical stimuli influence AQP4 alignment on astrocytic endfeet are not clearly defined. This gap in understanding represents a significant challenge, as it is crucial to determine whether such stimulation can be consistently effective across different brain regions and under various pathological conditions. Furthermore, the reported therapeutic effects are predominantly linked to the polarization and modulation of AQP4, with a focus on a single molecular mechanism. This narrow focus may have overlooked other critical biological processes involved in stroke recovery, such as neural activation, inflammation, neurogenesis, and synaptic plasticity, all of which are essential for comprehensive rehabilitation. Second, the PLA MNA for a hybrid electro‐optical stimulator is produced by replicating clinical acupuncture needles owing to its suitable geometric characteristics and availability. However, the proposed MNA fabrication process is compatible with various types of 3D microstructures because of the simple and highly effective double‐casting of thermoplastics. Consequently, microneedle structures can be further optimized in terms of the diameter, length, spacing, and number of channels. The current PLA MNA consisted of five pairs of microneedles and µLEDs. While the center channel is electrically and optically isolated from its surroundings, the four peripheral channels are collectively controlled, allowing simultaneous electro‐optical stimulation through the center and surrounding channels during animal experiments. Despite the effectiveness demonstrated in this study, the ability to individually address each of the five channels is expected to contribute to more localized and precise activation of the associated brain networks. Interconnections for individual electro‐optical stimulation can be readily achieved by increasing the number of wires and vias without compromising the electrical, mechanical, and electrochemical performance.

## 4 Conclusions

This study concluded that hybrid electro‐optical stimulation, by specifically targeting and modulating AQP4 polarization, has significant potential for enhancing glymphatic function and overall recovery from ischemic brain injuries. These findings underscore the potential of brain stimulation strategies focused on glymphatic drainage as a therapeutic approach, potentially revolutionizing treatment modalities for stroke recovery.

## 5 Experimental Section

### Fabrication of Hybrid Electro‐optical Stimulator

The entire fabrication process for the hybrid electro‐optical stimulator based on the PLAMNA is illustrated in Figure S5 (Supporting Information), along with sample photographs in Figure S6 (Supporting Information). (a) The master microneedle mold was produced by inserting five 0.3‐mm‐diameter acupuncture needles (DB107, Dongbang Medical, Korea) through two perforated metal sheets separated by 29 mm (Figure S6a,b, Supporting Information). (b) The PDMS mixture (10:1; Sylgard184, Dow) was poured on top of the lower metal sheet up to a thickness of 1 mm for fixation of the needles, which was cured at 80 °C for 2 h in an oven. (c) During this step, the capillary force and surface tension of the PDMS resin create meniscus‐like pedestals around the bottom of the microneedles, which provide mechanical support to the PLA microneedles against lateral stress. (d) The master needle mold was assembled into an aligned metal jig (Figure S7, Supporting Information) and precisely dipped into the PDMS mixture poured into a metal bath, followed by curing at room temperature for 48 h. (e) PDMS‐negative mold after removal of the master mold (Figure S6c, Supporting Information). (f) The PLA MNA was produced by melting PLA pellets (PLA, Corbion) on the PDMS‐negative mold in a vacuum oven at 220 °C for 1 h. (g, h) Subsequently, it was pressed by a structured metal plate with 0.3 mm of clearance, which created underside pockets for housing µLEDs (Figure S6d, Supporting Information). (i) The PLA MNA was drilled by two 0.5 mm‐diameter via holes, and (j, k) the top surface was selectively masked by patterned polyimide tape to electrically separate the center and surrounding needles during 300‐nm‐thick indium tin oxide (ITO) deposition (Figure S6e,f, Supporting Information). (l) Finally, the PLA MNA was individually diced using a CO_2_ laser. (m, n) A FPCB cable for delivering stimulation current and µLED power was assembled with five 850 nm µLEDs (15404085BA470, Wurth Elektronik), each measuring 1 × 0.5 × 0.5 mm, using conductive epoxy (8331D, MG chemicals). (o) After filling UV‐curable optical epoxy (NOA 84, NORLAND) into the µLED slots, the MNA was attached onto the µLED‐mounted FPCB and cured under a UV lamp for 5 min. (p) Silver epoxy was injected into the MNA to form electrical interconnections between the FPCB and ITO layers to deliver stimulation currents. (q) The PDMS mixture was spin‐coated on top of the ITO layer at 2000 rpm for 30 s to create a 25‐µm‐thick insulating layer, (r) before applying additional epoxy insulation on the side surfaces of the device.

### Characterization of the Hybrid Electro‐optical Stimulator

The light intensity was measured using an optical power meter (PM100D and S121C, Thorlabs) placed 3 mm away from the LEDs. The cumulative transmittance was assessed by sequentially adding layers (optical epoxy, PLA MNA, ITO, scalp, and skull) on top of the LED, while the transmittance of individual layers was computed by comparing the light intensity measured before and after each layer (Figure S3, Supporting Information).

The mechanical strength of the PLA microneedles under lateral stress was evaluated using a bending test. A force meter (F105, Mark 10) measured the bending force while pushing the midpoint of the needle at a speed of 0.25 mm s^−1^ using a 0.2‐mm‐wide metal tip.

MC simulation based on the photon packet method was used to compute the light transport from a µLED through a PLA microneedle using ValoMC, an open‐source MC code for MATLAB.^[^ ^32^ ^]^ The optical properties of the PLA were set as follows: refractive index = 1.45, absorption coefficient = 0.1 cm^−1^, scattering coefficient = 1 cm^−1^.

For computational simulations, the 3D surface model of the mouse brain was extracted from MRI scans, while the skin and skull models were extracted from CT images of the same animal using ITK‐SNAP software.^[^ ^33^ ^]^ A 3D volume mesh model was generated, consisting five components: scalp (0.33 S m^−1^), skull (0.0083 S m^−1^), CSF (1.79 S m^−1^), brain (0.33 S m^−1^), and electrodes.^[^ ^34^ ^]^ The current density, electric potential, and electric field on the cortex induced by tDCS were computed and visualized using a custom‐built application with a partial differential equation (PDE) toolbox in MATLAB 2022b (Mathworks, USA), by solving the following equation:

(1) $$-\nabla \cdot \left(\sigma \nabla V\right)=0$$

where *σ* is the electric conductivity, and *V* is the electric potential. Direct current (DC) conduction problems were solved by applying Dirichlet boundary conditions for *V* and Neumann boundary conditions for the surface current density, which represents the normal component of the current density on a face in 3D geometry.^[^ ^35^ ^]^

### Animal Experiments and Experimental Procedures

Male C57BL/6 mice (6 weeks old) were purchased from Hana Biotech (Ansan, Korea) and housed under 12‐h light/dark cycle with ad libitum access to food and water. The animal protocol for this study was approved by the Pusan National University Institutional Animal Care and Use Committee (PNU‐IACUC) under the ethical procedures and scientific standards (PNU‐2023‐0105). The minimum animal sample size was calculated using G*Power 3.1 software (Heinrich‐Heine‐Universität, Düsseldorf, Germany; http://www.gpower.hhu.de/). The sample size for each group was determined based on NSSs obtained from our pilot study results (Figure S10, Supporting Information; effect size *f* = 0.574, α = 0.05, and power = 0.8). Consequently, the sample size for behavioral testing was set to a minimum of nine animals per group. All experiments, including hybrid electro‐optical stimulation and sham‐operated stimulation, were conducted during the light phase of the animals' circadian cycle. Specifically, stimulations were administered between Zeitgeber Time (ZT) 2 and ZT 4, where ZT 0 corresponds to lights on and ZT 12 to lights off in the 12:12 light:dark cycle of our animal housing facility. The mice underwent daily sessions of hybrid electro‐optical stimulation, which included electrical (83.33 µA mm^−2^, charge density: 50 kC m^−2^) and optical (89 mW cm^−2^, 850 nm) stimulations on a heated pad at 37 °C starting 48 h after MCAO/R. Each session lasted for 10 min over 5 days. The stimulation needles were placed on hairless scalp, spaced 2 mm apart in both the ML and AP directions, with four needles positioned around the center needle (ML: 0, AP: +0.5 mm from the bregma) (Figure 1a,b). The reference position was placed over the skin of the neck as an extracephalic position using a needle (0.3 mm diameter, 50 mm long). To ensure consistency across experimental groups and eliminate potential confounding factors, all groups—including the model group and sham‐operated group—wore the hybrid electro‐optical stimulation device throughout the experimental period. However, only the treatment group received active stimulation. For pharmacological inhibition of AQP4, the mice received intraperitoneal injection of TGN‐020 (200 mg kg^−1^; #5425, Tocris Bioscience, Bristol, UK) dissolved in 20% SBE‐β‐CD (#HY‐17031, MedChemExpress, NJ, USA) 10 min after MCAO/R induction, as per the study conducted by Li et al.^[^ ^28^ ^]^

### Ischemic Stroke

The mice were subjected by MCAO/R as previously described.^[^ ^36^ ^]^ Briefly, the mice were anesthetized with 2% isoflurane in 20% O_2_ and 80% N_2_O. Following full anesthesia, a fiber‐optic probe was affixed to the exposed skull over the left MCA and regional cerebral blood flow (CBF) was continuously monitored using the PeriFlux Laser Doppler System 5000 (Perimed, Stockholm, Sweden) throughout the surgical and reperfusion phases. A silicon‐coated 7‐0 monofilament (#7019PK5Re, Duccol Corporation, Redlands, CA) was inserted into the internal carotid artery and subsequently advanced to occlude the MCA. The occlusion was maintained for 45 min, after which the filament was withdrawn to initiate reperfusion. Following suturing, the mice were allowed to recover under a heating lamp and were returned to their respective cages. The body temperature was maintained at 37.5 °C during surgery using a heating pad (Harvard Apparatus, Holliston, MA).

### Neurological Severity Scores

The NSSs were assessed at 7 and 14 d post‐MCAO/R using the following criteria: when mice were lifted by the tail, a score of 1 was assigned for flexion of the forelimb, 1 for flexion of the hindlimb, and 1 if the head moved >10° to the vertical axis within 30 s. Subsequently, the mice were placed on the floor and scored as follows: 0, normal movement; 1, inability to walk straight; 2, circling toward the paretic side; 3, falling onto the paretic side.

### MRI

MRI was performed using a 7.0 T MRI scanner (MRS7000, MR Solutions, UK). Mice were anesthetized with 2% isoflurane in 20% O_2_ and 80% N₂O for induction, and 1.5% isoflurane for maintenance. They were positioned in the MRI scanner in a prone position with a respiratory monitoring system in place. MRI scans were conducted before stimulation (on day 2) and on days 4, 6, and 16 after MCAO/R. The first scan (day 2) was performed to establish the initial lesion comparability between the MCAO/R group and the hybrid stimulation groups before the initiation of hybrid stimulation. Additional scans were performed on days 4, 6, and 16 to monitor infarction progression during and after the stimulation period. T2‐weighted imaging (T2WI) was acquired using a fast spin‐echo sequence (RARE) (TR/TE = 5000 ms/45 ms, FOV = 25 cm × 25 cm, slice thickness = 0.7 mm, interslice distance = 0.1 mm, number of slices = 22, number of averages = 3). MRI images were reconstructed using the VivoQuant version 5.2 software (Invicro, Needham, MA). The infarct volumes were quantified using the ImageJ software (Fiji, National Institutes of Health, Bethesda, MD). The infarct area was manually delineated in each brain slice, excluding the cerebellum, and the infarct volume was determined by summing the infarct areas across all slices. The average infarct volume for each group was then calculated to compare the MCAO/R and hybrid stimulation groups at different time points, allowing for evaluation of the impact of hybrid electro‐optical stimulation on infarction size reduction.

### Rotarod Test

The locomotor function of the mice was evaluated using the rotarod test, which measures the average latency until falling from a rotating rod (Panlab S.L.U., Barcelona, Spain). Prior to testing, the mice underwent pre‐training to adapt to the procedure. Subsequently, they were placed on a rotating rod at 18 rpm for a maximum of 3 min. Each mouse completed five trials, and the average latency was calculated.

### Wire Grip Test

The wire grip test was used to assess the vestibular motor function. Each mouse was suspended on a metallic wire and hung using both forepaws. The wire grip performance was graded as follows: 1 = not gripping the wire; 2 = gripping the wire with both forepaws and hind paws but not the tail; 3 = gripping the wire with both forepaws and hind paws and the tail without movement; 4 = moving along the wire using all limbs and tails; 5 = moving adeptly along the wire.^[^ ^37^ ^]^

### Y‐Maze Test

This test was commonly used to evaluate the spatial working memory in rodents. The mice were placed in a Y‐shaped maze featuring three identical arms (A, B, and C) diverging at 120° from the central point. The mice were allowed to explore the maze freely for 8 min while being recorded using a video tracking system (Panlab, Barcelona, Spain). When an animal selects an arm different from the one it initially enters, it is referred to as an alternation. Alternation is considered a correct response (e.g., CBABA), whereas revisiting the preceding arm is countered as an error (e.g., CBBCBACA). The percentage of spontaneous alternation was calculated using the following formula: alternations / (total arm entries − 2) × 100.

### Morris Water Maze

The Morris water maze is a widely used behavioral test for assessing spatial learning and memory in rodents. This test used a large circular pool (diameter: 120 cm; depth: 50 cm) filled with water and a submerged circular target platform (10 × 10 cm). The water temperature was maintained at 25 ± 1 °C. The mice were tested for seven consecutive days. On the first day, the mice were given a 90‐s trial during which they swam in the pool with a visible platform to aid acclimatization to the water. In each trial, the mice began swimming from a distinct quadrant of the pool while the position of the platform remained constant. The time taken to reach the platform (escape latency) was recorded, and the average of four trials was calculated. The mice that reached the platform within 90 s were allowed to remain there for 15 s. If the mouse failed to find the platform within 90 s, it was gently guided to the platform and allowed to remain there for 30 s. From days 2 to 6, the learning trial followed the same protocol as that on day 1; however, the pool was filled with opaque water (hidden platform). On day 7, a probe trial was conducted, during which the mice were allowed to swim freely for 90 s without a platform. SMART software (Panlab) was used to measure the percentage of time spent in the quadrant where the platform had been positioned and to record the movement.

### Glymphatic System Assessment

For the glymphatic system experiment evaluating CSF infusion, a solution of 0.5% bovine serum albumin conjugated with Alexa 647 (#A34785, Invitrogen, Carlsbad, CA) in artificial CSF (aCSF, #3525, Tocris, Bristol, UK) was injected into the cisterna, following previously described methods.^[^ ^38^ ^]^ Briefly, the mice were anesthetized with 2% isoflurane and fixed in a stereotaxic frame (#51730, Stoelting Co., Wood Dale, IL). The mice were positioned with a slight tilt of the head to achieve a 120° angle to their bodies, and the head and neck were shaved. Using the occipital crest as a reference point, the cisterna magna was exposed, and a prepared cannula (consisting of a 30 G dental needle inserted into PE10 tubing (Polyethylene Tubing 0.024 in. OD × 0.011 in. ID) filled with a CSF tracer) was inserted into the center of the cisterna magna, ≈1–2 mm deep. The CSF tracer was injected at a rate of 1 µL min^−1^ for 10 min using a syringe pump (KDS‐311, KD Scientific Inc., Holliston, MA). Approximately 30 min after the injection, the mice were perfused and decapitated to collect their brains.

To assess the CSF clearance, the anesthetized mice were positioned in a stereotaxic frame, and the scalp was incised to expose the skull. A solution of 0.5% BSA‐Alexa 647 conjugate was injected into the striatum (AP: +0.5, ML: +2, DV: −3 mm from bregma) at a rate of 0.2 µL min^−1^ for 10 min using a 10 µL syringe and a 33 G needle (Hamilton, Bonaduz, Switzerland). After the injection, the needle was left in place for 10 min before being slowly withdrawn to prevent CSF leakage. Two hours after the injection, the mice were perfused and decapitated to collect their brains. To assess the glymphatic system, a whole‐slice montage of seven brain sections per animal was captured using an EDF stitch array with a K‐1 Fluo microscope (Nanoscope Systems, Daejeon, Korea) at 10× objective power. The mean intensity or fluorescent area coverage of the fluorescent tracer within each section was measured using ImageJ (National Institutes of Health).

### Histology and Immunofluorescence Staining

The mice were perfused with cold PBS followed by 4% paraformaldehyde (PFA). Subsequently, the brains were harvested and further fixed for 24 h in 4% paraformaldehyde, followed by cryoprotection in 30% sucrose for 72 h at 4°C. Each brain was then embedded in optical cutting temperature (OCT) compound (Sakura Finetek, Torrance, CA) and stored at −80 °C until analysis. The frozen brains were sectioned into 40 µm thick slices using a cryostat (CM 3050, Leica Microsystems, Wetzlar, Germany).

Nissl staining was used to assess the atrophy volume. The frozen sections were rehydrated using descending alcohol series (100%, 90%, 80%, 70%, and 50%) and distilled water. The brain sections were then incubated in a 0.1% cresyl violet solution (Sigma‐Aldrich, St. Louis, MO) for 15 min, and rinsed in distilled water. After differentiation in 95% ethanol for 5 min and dehydration in 100% ethanol for 10 min twice, the sections were cleared with xylene for 5 min and mounted using a permanent mounting medium (H‐5000, Vector Laboratories, Newark, CA). For immunofluorescence staining, the brain sections were immunostained with Aquaporin‐4 (AQP‐4, 1:200, AB3594, Millipore, Billerica, MA), glial fibrillary acidic protein (GFAP, 1:200, MAB360, Millipore), CD31 (1:100, 550274, BD Biosciences, Franklin Lakes, NJ), IL‐1β (1:100, AF‐401, R&D Systems, Minneapolis, MN), cleaved caspase‐3 (1:100, 9661S, Cell Signaling, Danvers, MA), Iba1 (1:200, 019‐19741, Wako, Osaka, Japan), NeuN (1:100, MAB377, ABN78, Millipore), Ki67 (1:200, ab15580, Cambridge, UK), and DCX (1:100, sc‐8066, Santa Cruz, Dallas, TX) antibodies overnight at 4 °C. Subsequently, the cells were incubated with Alexa 488‐(1:500, A‐11001, A‐11006, A‐11055; Life Technologies, Carlsbad, CA) or Alexa 594‐(1:500, A‐11005, A‐11037; Life Technologies) conjugated secondary antibodies for 2 h in the dark. The nuclei were stained using 4′, 6‐diamidino‐2‐phenylindole (DAPI, Molecular Probes), and the sections were mounted with a mounting solution (H‐1400, Vector Laboratories).

### Image Analysis

All image analyses were performed using the ImageJ software. Nissl‐stained sections were observed under a microscope (Stemi 305, Carl Zeiss, Jena, Germany), and atrophic volume was calculated as the ratio of ipsilateral to contralateral volume multiplied by 100. The fluorescence images were captured at 20× and 40× objective power using K‐1 Fluo microscope (Nanoscope Systems). To quantify the regional AQP4, CD31, NeuN, and GFAP expression, a threshold was uniformly applied and the immunoreactivity‐positive area was measured as a percentage of the entire regional area.

The quantification of AQP4 polarization was performed as previously described.^[^ ^39^ ^]^ Briefly, the DAPI^+^GFAP^+^AQP^+^ merged images were segmented into AQP4 single channels and DAPI/GFAP channels. The global AQP4 fluorescence intensity (FI) was measured as the mean intensity within the AQP4 single channel. Empty spaces enclosed by GFAP and elliptical nuclei as capillaries were identified and then donut‐shaped areas in the DAPI/GFAP channels were drawn. The regions of interest (ROIs) were applied to the same location on the AQP4 channel. The AQP4 polarization index was calculated as the ratio between the average FI of perivascular AQP4 and its overall distribution (Figure S15, Supporting Information). Thirty vessels were analyzed per group.

### Western Blotting

Protein was isolated from brain tissues using standard techniques and lysed with radioimmunoprecipitation assay (RIPA) buffer (9806, Cell Signaling), and then supplemented with a protease inhibitor mixture (GenDEPOT, Katy, TX) and a phosphatase inhibitor mixture (GenDEPOT). Equal amounts of total protein (50 µg) were separated using 12% sodium dodecyl sulfate‐polyacrylamide gel electrophoresis (SDS‐PAGE), transferred onto a nitrocellulose membrane (10600004, Amersham, Little Chalfort, UK) or a polyvinylidene difluoride (PVDF) membrane (10600023, Amersham), and immunoblotted with antibodies specific to IL‐1β (1:1000, ab234437, Abcam), caspase‐3 (1:1000, 9662, Cell Signaling), and cleaved caspase‐3 (1:1000, 9661S, Cell Signaling) overnight at 4 °C. α‐tubulin (1:5000, A5316, Sigma‐Aldrich) was used to confirm equal protein loading. Horseradish peroxidase‐conjugated goat anti‐rabbit (1:4000, 4050‐05, Southern Biotech, Birmingham, AL) and anti‐mouse (1:4000, 1031‐05, Southern Biotech) were used as secondary antibodies. The immunoblots were incubated for 1 h. The intensity of chemiluminescence was measured using an ImageQuant LAS 4000 apparatus (GE Healthcare Life Sciences, Uppsala, Sweden), and band intensity was quantified using ImageJ (National Institutes of Health).

### Statistical Analysis

Data are expressed as mean ± standard error of the mean (SEM) in all figures. For normally distributed data with equal variances, one‐way analysis of variance (ANOVA) was performed, followed by Tukey's post‐hoc test for multiple group comparisons. The sample size (*n*) for each experiment is indicated in the figure legends, and the P values are described in the results section. Statistical significance was defined as *p* < 0.05. All statistical analyses were conducted using SigmaPlot 12.5 (Systat Software Inc, San Jose, CA).

## Conflict of Interest

The authors declare no conflict of interest.

## Author Contributions

M.J.K. and J.Y. contributed equally to this work. B.T.C., Y.‐I.S., J.J., and H.K.S. participated in research design. M.J.K., J.Y., H.J.L., and T.G.K. conducted experiments. S.‐Y.L., Y.‐J.J., and Y.‐I.S. contributed new reagents or analytic tools. M.J.K., J.Y., J.J., and H.K.S. performed data analysis. M.J.K., J.Y., J.J., and H.K.S. wrote or contributed to the writing of the manuscript.

## Supporting information



Supporting Information

## Data Availability

Research data are not shared.

## References

[1] J. N. Stankowski , R. Gupta , Antioxid. Redox Signaling 2011, 14, 1841. 10.1089/ars.2010.3292 PMC3120088 20626319

[2] S. S. Martin , A. W. Aday , Z. I. Almarzooq , C. A. M. Anderson , P. Arora , C. L. Avery , C. M. Baker‐Smith , B. Barone Gibbs , A. Z. Beaton , A. K. Boehme , Y. Commodore‐Mensah , M. E. Currie , M. S. V. Elkind , K. R. Evenson , G. Generoso , D. G. Heard , S. Hiremath , M. C. Johansen , R. Kalani , D. S. Kazi , D. Ko , J. Liu , J. W. Magnani , E. D. Michos , M. E. Mussolino , S. D. Navaneethan , N. I. Parikh , S. M. Perman , R. Poudel , M. Rezk‐Hanna , et al., Circulation 2024, 149, e347. 38264914 10.1161/CIR.0000000000001209 PMC12146881

[3] a) J. Anrather , C. Iadecola , Neurotherapeutics 2016, 13, 661; 27730544 10.1007/s13311-016-0483-x PMC5081118

[4] E. Esposito , B. J. Ahn , J. Shi , Y. Nakamura , J. H. Park , E. T. Mandeville , Z. Yu , S. J. Chan , R. Desai , A. Hayakawa , X. Ji , E. H. Lo , K. Hayakawa , Nat. Commun. 2019, 10, 5306. 31757960 10.1038/s41467-019-13324-w PMC6876639

[5] D. B. Back , K. J. Kwon , D. H. Choi , C. Y. Shin , J. Lee , S. H. Han , H. Y. Kim , J. Neuroinflammation 2017, 14, 216. 29121965 10.1186/s12974-017-0992-5 PMC5679180

[6] a) W. Peng , T. M. Achariyar , B. Li , Y. Liao , H. Mestre , E. Hitomi , S. Regan , T. Kasper , S. Peng , F. Ding , H. Benveniste , M. Nedergaard , R. Deane , Neurobiol. Dis. 2016, 93, 215; 27234656 10.1016/j.nbd.2016.05.015 PMC4980916

[7] J. J. Iliff , M. Wang , Y. Liao , B. A. Plogg , W. Peng , G. A. Gundersen , H. Benveniste , G. E. Vates , R. Deane , S. A. Goldman , E. A. Nagelhus , M. Nedergaard , Sci. Transl. Med. 2012, 4, 147ra111. 10.1126/scitranslmed.3003748 PMC3551275 22896675

[8] a) H. Mestre , L. M. Hablitz , A. L. Xavier , W. Feng , W. Zou , T. Pu , H. Monai , G. Murlidharan , R. M. Castellanos Rivera , M. J. Simon , M. M. Pike , V. Plá , T. Du , B. T. Kress , X. Wang , B. A. Plog , A. S. Thrane , I. Lundgaard , Y. Abe , M. Yasui , J. H. Thomas , M. Xiao , H. Hirase , A. Asokan , J. J. Iliff , M. Nedergaard , Elife 2018, 7, e40070. 30561329 10.7554/eLife.40070 PMC6307855

[9] J. Zhu , J. Mo , K. Liu , Q. Chen , Z. Li , Y. He , Y. Chang , C. Lin , M. Yu , Y. Xu , X. Tan , K. Huang , S. Pan , Stroke 2024, 55, 1393. 38533660 10.1161/STROKEAHA.123.045941

[10] T. Lv , B. Zhao , Q. Hu , X. Zhang , Front. Aging Neurosci. 2021, 13, 689098. 34305569 10.3389/fnagi.2021.689098 PMC8297504

[11] W. K. Ting , F. A. Fadul , S. Fecteau , C. Ethier , Front. Neurosci. 2021, 15, 649459. 34054410 10.3389/fnins.2021.649459 PMC8160247

[12] a) J. A. Zivin , G. W. Albers , N. Bornstein , T. Chippendale , B. Dahlof , T. Devlin , M. Fisher , W. Hacke , W. Holt , S. Ilic , S. Kasner , R. Lew , M. Nash , J. Perez , M. Rymer , P. Schellinger , D. Schneider , S. Schwab , R. Veltkamp , M. Walker , J. Streeter , Stroke 2009, 40, 1359; 19233936 10.1161/STROKEAHA.109.547547

[13] a) A. H. Javadi , V. Walsh , Brain Stimul. 2012, 5, 231; 21840287 10.1016/j.brs.2011.06.007

[14] a) H. I. Lee , S. W. Lee , N. G. Kim , K. J. Park , B. T. Choi , Y. I. Shin , H. K. Shin , J. Biophotonics 2017, 10, 1502; 28164443 10.1002/jbio.201600244

[15] a) A. Liu , M. Vöröslakos , G. Kronberg , S. Henin , M. R. Krause , Y. Huang , A. Opitz , A. Mehta , C. C. Pack , B. Krekelberg , A. Berényi , L. C. Parra , L. Melloni , O. Devinsky , G. Buzsáki , Nat. Commun. 2018, 9, 5092; 30504921 10.1038/s41467-018-07233-7 PMC6269428

[16] A. Antal , I. Alekseichuk , M. Bikson , J. Brockmöller , A. R. Brunoni , R. Chen , L. G. Cohen , G. Dowthwaite , J. Ellrich , A. Flöel , F. Fregni , M. S. George , R. Hamilton , J. Haueisen , C. S. Herrmann , F. C. Hummel , J. P. Lefaucheur , D. Liebetanz , C. K. Loo , C. D. McCaig , C. Miniussi , P. C. Miranda , V. Moliadze , M. A. Nitsche , R. Nowak , F. Padberg , A. Pascual‐Leone , W. Poppendieck , A. Priori , S. Rossi , et al., Clin. Neurophysiol. 2017, 128, 1774. 28709880 10.1016/j.clinph.2017.06.001 PMC5985830

[17] D. H. Jung , J. H. Lee , H. J. Lee , J. W. Park , Y. J. Jung , H. K. Shin , B. T. Choi , Theranostics 2024, 14, 1325. 38389833 10.7150/thno.90779 PMC10879864

[18] a) J. Y. Han , J. H. Kim , J. H. Park , M. Y. Song , M. K. Song , D. J. Kim , Y. N. You , G. C. Park , J. B. Choi , M. R. Cho , J. C. Shin , J. H. Cho , Trials 2016, 17, 490; 27724972 10.1186/s13063-016-1611-y PMC5057263

[19] M. P. Jackson , A. Rahman , B. Lafon , G. Kronberg , D. Ling , L. C. Parra , M. Bikson , Clin. Neurophysiol. 2016, 127, 3425. 27693941 10.1016/j.clinph.2016.08.016 PMC5083183

[20] M. A. Moskowitz , E. H. Lo , C. Iadecola , Neuron 2010, 67, 181. 20670828 10.1016/j.neuron.2010.07.002 PMC2957363

[21] L. Lin , X. Hao , C. Li , C. Sun , X. Wang , L. Yin , X. Zhang , J. Tian , Y. Yang , J. Stroke Cerebrovasc. Dis. 2020, 29, 104828. 32404284 10.1016/j.jstrokecerebrovasdis.2020.104828

[22] H. Mestre , T. Du , A. M. Sweeney , G. Liu , A. J. Samson , W. Peng , K. N. Mortensen , F. F. Stæger , P. A. R. Bork , L. Bashford , E. R. Toro , J. Tithof , D. H. Kelley , J. H. Thomas , P. G. Hjorth , E. A. Martens , R. I. Mehta , O. Solis , P. Blinder , D. Kleinfeld , H. Hirase , Y. Mori , M. Nedergaard , Science 2020 , 367, eaax7171. 32001524 10.1126/science.aax7171 PMC7375109

[23] a) L. Xie , H. Kang , Q. Xu , M. J. Chen , Y. Liao , M. Thiyagarajan , J. O'Donnell , D. J. Christensen , C. Nicholson , J. J. Iliff , T. Takano , R. Deane , M. Nedergaard , Science 2013, 342, 373; 24136970 10.1126/science.1241224 PMC3880190

[24] O. Semyachkina‐Glushkovskaya , T. Penzel , I. Blokhina , A. Khorovodov , I. Fedosov , T. Yu , G. Karandin , A. Evsukova , D. Elovenko , V. Adushkina , A. Shirokov , A. Dubrovskii , A. Terskov , N. Navolokin , M. Tzoy , V. Ageev , I. Agranovich , V. Telnova , A. Tsven , J. Kurths , Cells 2021, 10, 3289. 34943796 10.3390/cells10123289 PMC8699220

[25] Y. Lee , Y. Choi , E. J. Park , S. Kwon , H. Kim , J. Y. Lee , D. S. Lee , Sci. Rep. 2020, 10, 16144. 32999351 10.1038/s41598-020-73151-8 PMC7527457

[26] Y. Lin , J. Jin , R. Lv , Y. Luo , W. Dai , W. Li , Y. Tang , Y. Wang , X. Ye , W. J. Lin , Acta Neuropathol. Commun. 2021, 9, 102. 34078467 10.1186/s40478-021-01198-3 PMC8170932

[27] X. Zhou , Y. Li , C. Lenahan , Y. Ou , M. Wang , Y. He , Front. Aging Neurosci. 2021, 13, 698036. 34421575 10.3389/fnagi.2021.698036 PMC8372556

[28] X. Li , Z. Xie , Q. Zhou , X. Tan , W. Meng , Y. Pang , L. Huang , Z. Ding , Y. Hu , R. Li , G. Huang , H. Li , Mol. Neurobiol. 2024, 61, 1175. 37695472 10.1007/s12035-023-03636-w PMC10861636

[29] G. T. Manley , M. Fujimura , T. Ma , N. Noshita , F. Filiz , A. W. Bollen , P. Chan , A. S. Verkman , Nat. Med. 2000, 6, 159. 10655103 10.1038/72256

[30] X. N. Zeng , L. L. Xie , R. Liang , X. L. Sun , Y. Fan , G. Hu , CNS Neurosci. Ther. 2012, 18, 388. 22533723 10.1111/j.1755-5949.2012.00308.x PMC6493383

[31] W. Z. Shi , C. Z. Zhao , B. Zhao , Q. J. Shi , L. H. Zhang , Y. F. Wang , S. H. Fang , Y. B. Lu , W. P. Zhang , E. Q. Wei , Neurosci. Bull. 2012, 28, 680. 23132680 10.1007/s12264-012-1281-z PMC5561818

[32] A. A. Leino , A. Pulkkinen , T. Tarvainen , OSA Continuum 2019, 2, 957.

[33] P. A. Yushkevich , J. Piven , H. C. Hazlett , R. G. Smith , S. Ho , J. C. Gee , G. Gerig , Neuroimage 2006, 31, 1116. 16545965 10.1016/j.neuroimage.2006.01.015

[34] W. L. Barnes , W. H. Lee , A. V. Peterchev , Annu. Int. Conf. IEEE Eng. Med. Biol. Soc. 2014, 2014, 6129. 25571396 10.1109/EMBC.2014.6945028

[35] A. H. D. Cheng , D. T. Cheng , Eng. Anal. Boundary Elem. 2005, 29, 268.

[36] H. Kim , J. S. Seo , S. Y. Lee , K. T. Ha , B. T. Choi , Y. I. Shin , Y. J. Yun , H. K. Shin , Brain, Behav., Immun. 2020, 87, 765. 32201254 10.1016/j.bbi.2020.03.011

[37] M. J. Kim , K. H. Park , J. Y. Lee , K. T. Ha , B. T. Choi , J. U. Baek , Y. J. Yun , S. Y. Lee , H. K. Shin , Oxid. Med. Cell. Longevity 2019, 2019, 4379732. 10.1155/2019/4379732 PMC6914926 31885791

[38] a) A. L. R. Xavier , N. L. Hauglund , S. von Holstein‐Rathlou , Q. Li , S. Sanggaard , N. Lou , I. Lundgaard , M. Nedergaard , J. Vis. Exp. 2018, 10.3791/57378. PMC6101354 29889209

[39] J. Cao , D. Yao , R. Li , X. Guo , J. Hao , M. Xie , J. Li , D. Pan , X. Luo , Z. Yu , M. Wang , W. Wang , Neurosci. Bull. 2022, 38, 181. 34704235 10.1007/s12264-021-00772-y PMC8821764

[40] a) Z. Chen , J. H. C. Lai , J. Xu , H. Zhang , J. Huang , K. W. Y. Chan , NMR Biomed. 2024, 37, e5093; 38163739 10.1002/nbm.5093

[41] I. Pirici , T. A. Balsanu , C. Bogdan , C. Margaritescu , T. Divan , V. Vitalie , L. Mogoanta , D. Pirici , R. O. Carare , D. F. Muresanu , Int. J. Mol. Sci. 2017, 19, 46. 29295526 10.3390/ijms19010046 PMC5795996

[42] L. M. Hablitz , V. Plá , M. Giannetto , H. S. Vinitsky , F. F. Stæger , T. Metcalfe , R. Nguyen , A. Benrais , M. Nedergaard , Nat. Commun. 2020, 11, 4411. 32879313 10.1038/s41467-020-18115-2 PMC7468152

[43] S. Yang , C. Qin , M. Chen , Y. H. Chu , Y. Tang , L. Q. Zhou , H. Zhang , M. H. Dong , X. W. Pang , L. Chen , L. J. Wu , D. S. Tian , W. Wang , Adv. Sci. 2024, 11, e2305614. 10.1002/advs.202305614 PMC10933614 38151703

