# Supplementary material for: Hybrid Electro‐optical Stimulation Improves Ischemic Brain Damage by Augmenting the Glymphatic System
Source: Adv Sci (Weinh). 2025 Feb 10;12(13):2417449. doi: 10.1002/advs.202417449 (PMC11967803; doi:10.1002/advs.202417449)

**Supporting Information**

**Hybrid Electro-optical Stimulation Improves Ischemic Brain Damage by Augmenting the Glymphatic System**

*Min Jae Kim, Jiman Youn, Hong Ju Lee, Seo-Yeon Lee, Tae Gue Kim, Young-Jin Jung, Yong-Il Shin, Byung Tae Choi, Joonsoo Jeong*, Hwa Kyoung Shin**

H. J. Kim, H. J. Lee, B. T. Choi, Prof. H. K. Shin

Department of Korean Medical Science

School of Korean Medicine

Pusan National University

Yangsan, Gyeongnam 50612, Republic of Korea

E-mail: julie@pusan.ac.kr (H. K. S.)

H. J. Kim, H. J. Lee, B. T. Choi, Prof. H. K. Shin

Graduate Training Program of Korean Medical Therapeutics for Healthy-Aging

Pusan National University

Yangsan, Gyeongnam 50612, Republic of Korea

J. Youn, J. Jeong

Department of Information Convergence Engineering

Pusan National University

Yangsan 50612, Republic of Korea

E-mail: joonsoo_jeong@pusan.ac.kr (J. J.)

S.-Y. Lee

Department of Pharmacology

Wonkwang University School of Medicine

Iksan, 54538, Republic of Korea

T. G. Kim, Y.-J. Jung

School of Healthcare and Biomedical Engineering

Chonnam National University

Yeosu 59626, Republic of Korea

Y.-I. Shin

Department of Rehabilitation Medicine

School of Medicine, Pusan National University

Yangsan, Gyeongnam 50612, Republic of Korea

J. Jeong

School of Biomedical Convergence Engineering

Pusan National University

Yangsan 50612, Republic of Korea

Keywords: Hybrid electro-optical stimulation, Glymphatic system, Cerebrospinal fluid, Ischemic stroke, Aquaporin-4 polarization

**Supplementary method**

**Photothrombotic cortical ischemia**

The mice were anesthetized with 2% isoflurane in 20% O_2_ and 80 %N_2_O. Subsequently, they received an intraperitoneal (i.p.) injection of Rose Bengal (Sigma-Aldrich, St. Louis, MO; 0.1 mL of 10 mg/mL in 0.9% saline) 5 min prior to illumination. Each mouse was fixed on a stereotaxic frame (David Kopf Instruments, Tujunga, CA, USA) and the skull was exposed. A fiber-optic bundle containing a KL 6000 LED cold light source (Carl Zeiss, Jena, Germany) was positioned on the sensorimotor cortex of the exposed skull (2.4 mm lateral to bregma) and illuminated for 15 min. The scalp was sutured after illumination. Subsequently, the mice were allowed to recover under a heating lamp and were returned to their home cages. The body temperature was maintained at 37.5°C during surgery using a heating pad (Harvard Apparatus, Holliston, MA).

**RNA-seq**

For RNA-seq analysis, four mice from each group (control, MCAO/R, and hybrid) were selected based on their performance in the wire grip test following ES after MCAO. The selected mice were anesthetized with sodium pentobarbital (50 mg/kg, SCI Pharmtech Inc., Taoyuan, Taiwan), and the cortical peri-infarct regions of their brains were quickly isolated for RNA-seq at Ebiogen Inc. (Seoul, Republic of Korea). Total RNA was extracted from the peri-infarct region of the cortex using TRIzol reagent (Invitrogen, Carlsbad, CA, USA). Quality control of the raw sequencing data was performed using the FastQC software to ensure high-quality reads. The RNA libraries were prepared using the QuantSeq 3′ mRNA-Seq Library Prep Kit FWD (Lexogen, Vienna, Austria) following the manufacturer’s instructions, and purified libraries were quantified using a Qubit 2.0 Fluorometer and Agilent 2100 Bioanalyzer. High-throughput single-end 75 sequencing was performed on a NextSeq 500/550 system (Illumina, San Diego, CA, USA). Differentially expressed genes (DEGs) were identified using a threshold of fold change > 2.0, normalized data (log2) > 4, and a P-value < 0.05 for statistical significance. DEGs and GO analyses were performed using an Excel-based DEG analysis software package (ExDEGA, Ebiogen Inc., Seoul, Republic of Korea). The Database for Annotation, Visualization, and Integrated Discovery (DAVID, https://david.ncifcrf.gov/) was used to perform KEGG pathway enrichment and GO functional analyses. GO terms were considered significant when the false discovery rate (FDR)-adjusted P-value was < 0.05.

**Figure S1.** (a) Photograph of transparent conductive microneedle array (MNA) assembled with µLED-mounted FPCB board for hybrid electro-optical stimulation, and (b) its schematic modeling including wired interconnection.


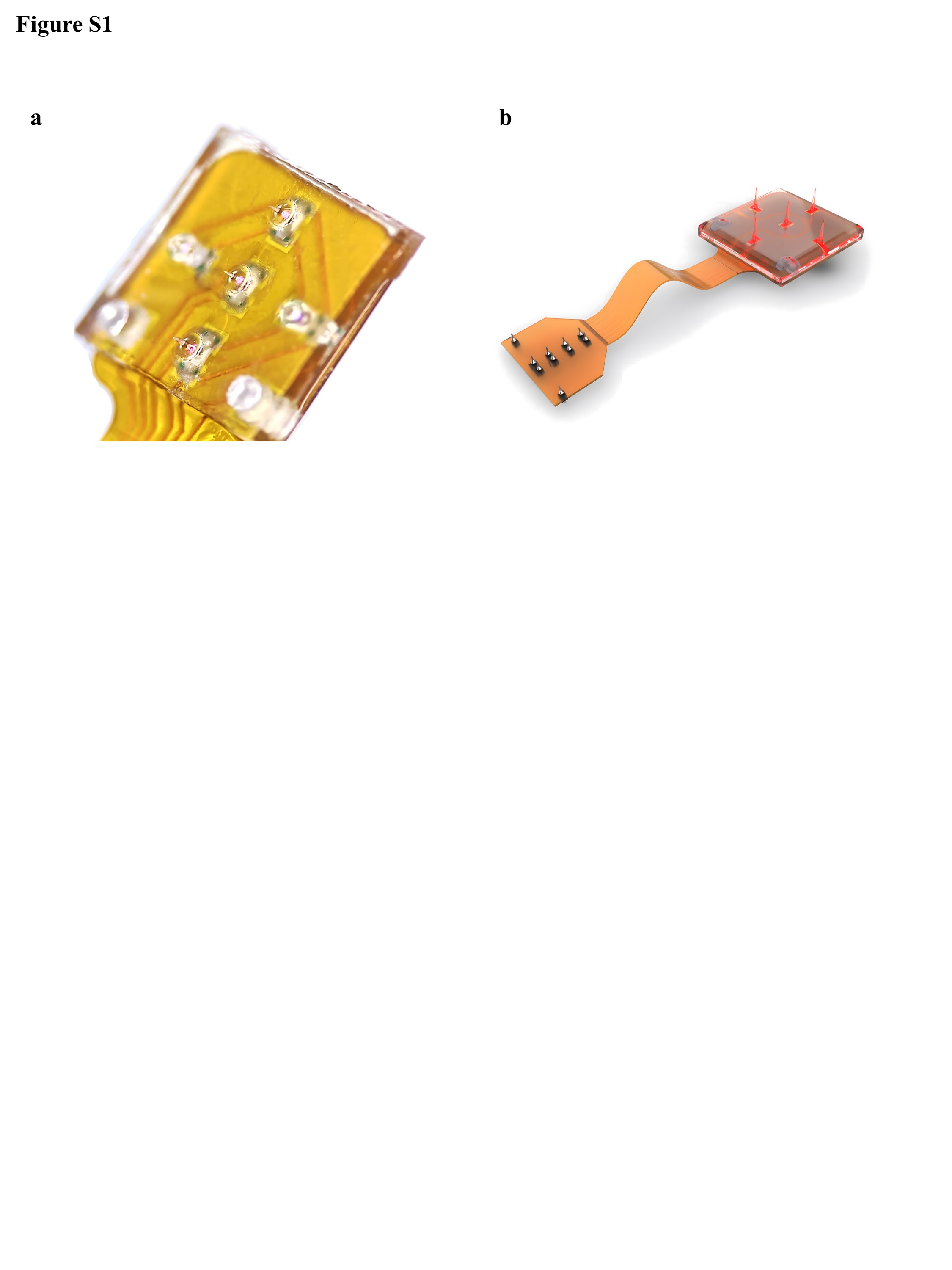


**Figure S2.** (a) Circuit layout of the FPCB cable of the hybrid electro-optical stimulator. (b) Magnified view of the stimulator area (dashed box in (a)), including contact pads and interconnections for five LEDs, and contact pads for supplying current pulses. Abbreviations: Flexible printed circuit board (FPCB), Light-emitting diode (LED), Ground (GND).


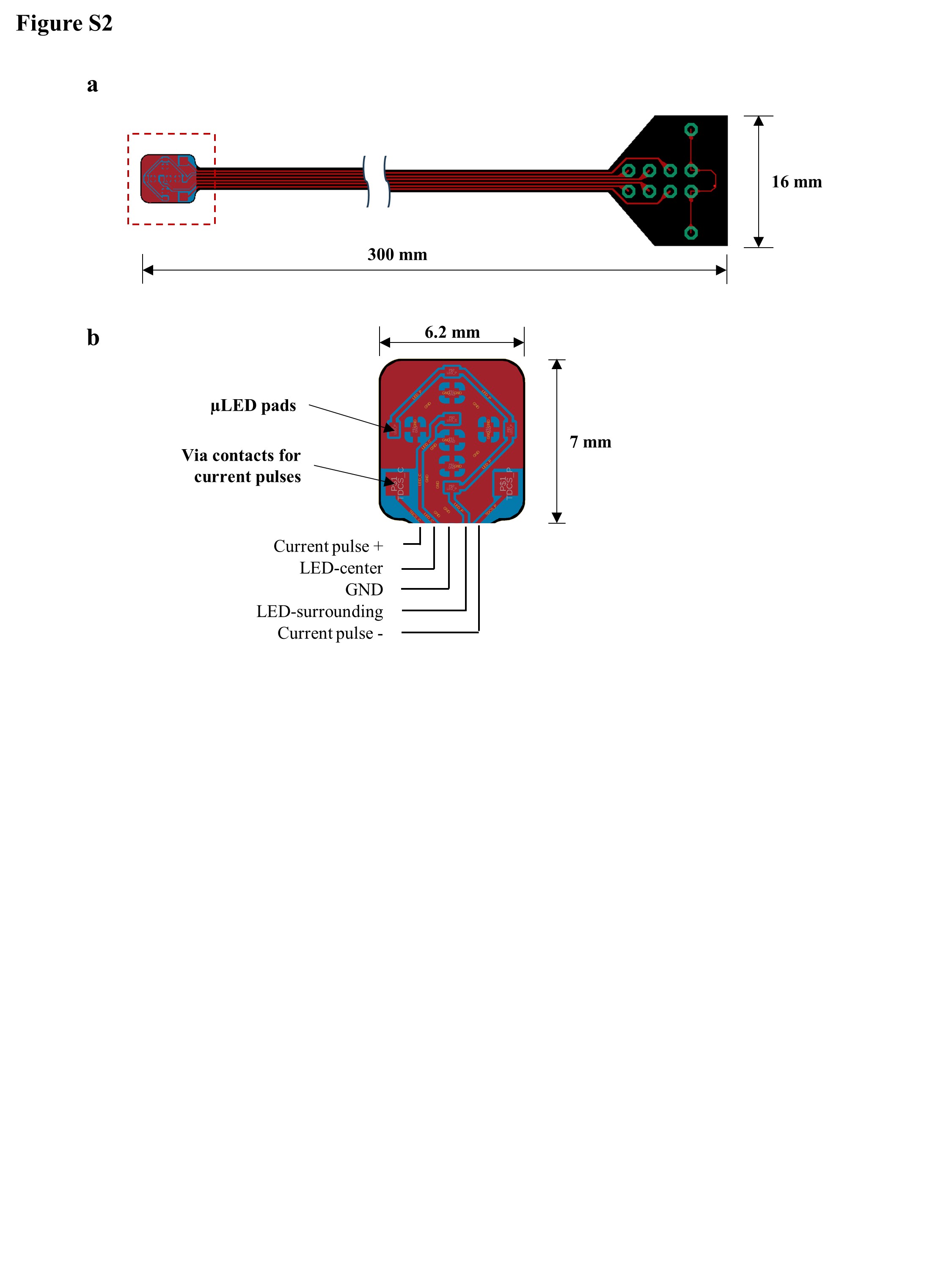


**Figure S3.** Experimental setup for measuring the cumulative transmittance of the layers used for PLA MNA. (a) Optical power was measured by sequentially adding layers on top of 850 nm µLEDs: i) µLEDs only, ii) optical epoxy added, iii) (ii) PLA MNA added, iv) scalp added, and v) skull added. Photographs with white light (left) and IR filter (right) of the tested PLA MNA for optical power measurements: (b) at step (iv) with the mouse scalp (left) and (c) at step (v) with the skull. Abbreviations: Microlight-emitting diodes (μLED).


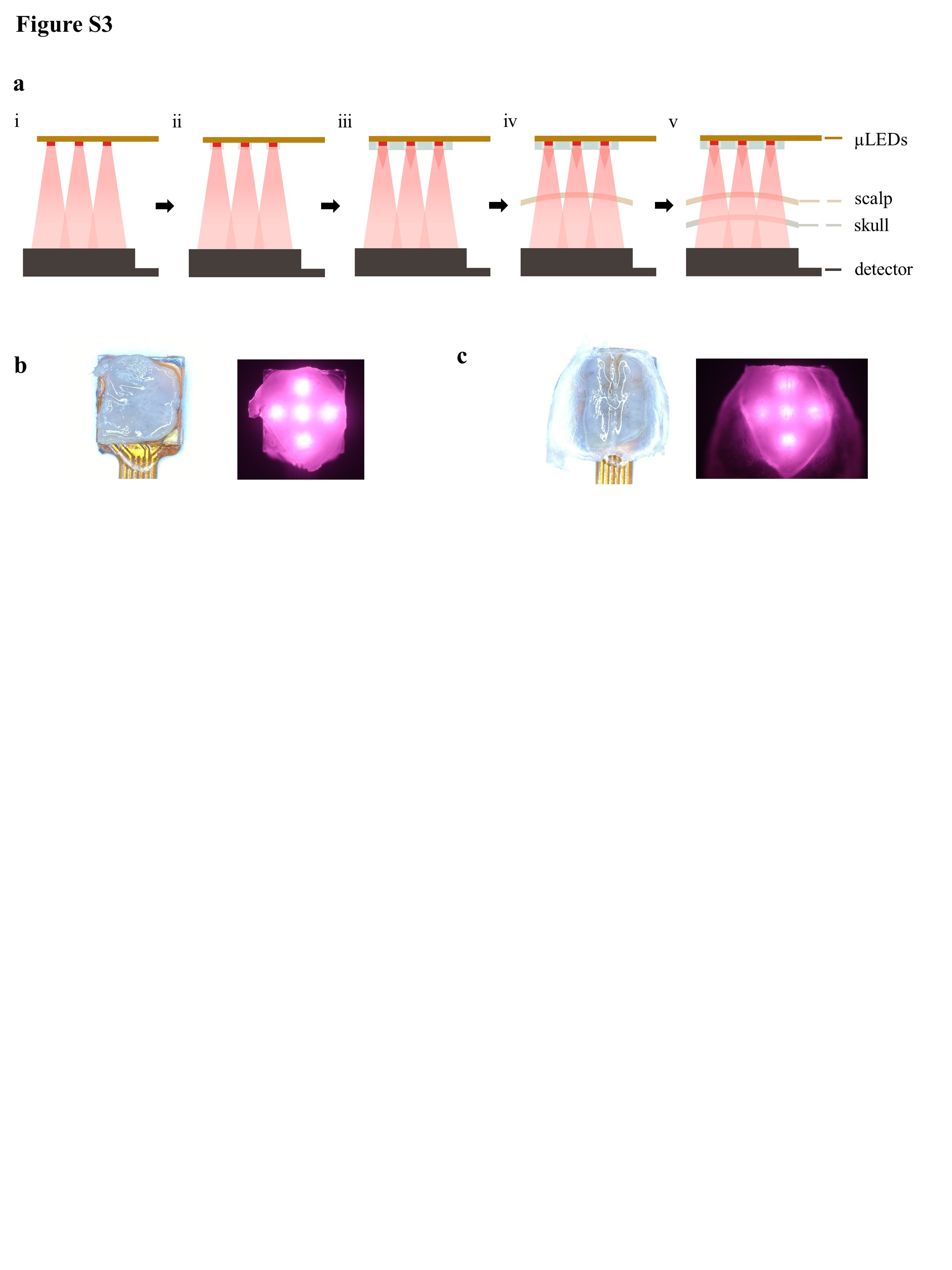


**Figure S4.** Skull thickness and optical transmittance of mice with varying ages of 6, 26, and 43 weeks. (a) Photographs of skulls from each age, along with illustration of thickness measurement points relative to center of stimulation. (b) Variation in skull thickness at five different points (*t*_1_–*t*_5_) and overall optical transmittance as a function of mouse age.


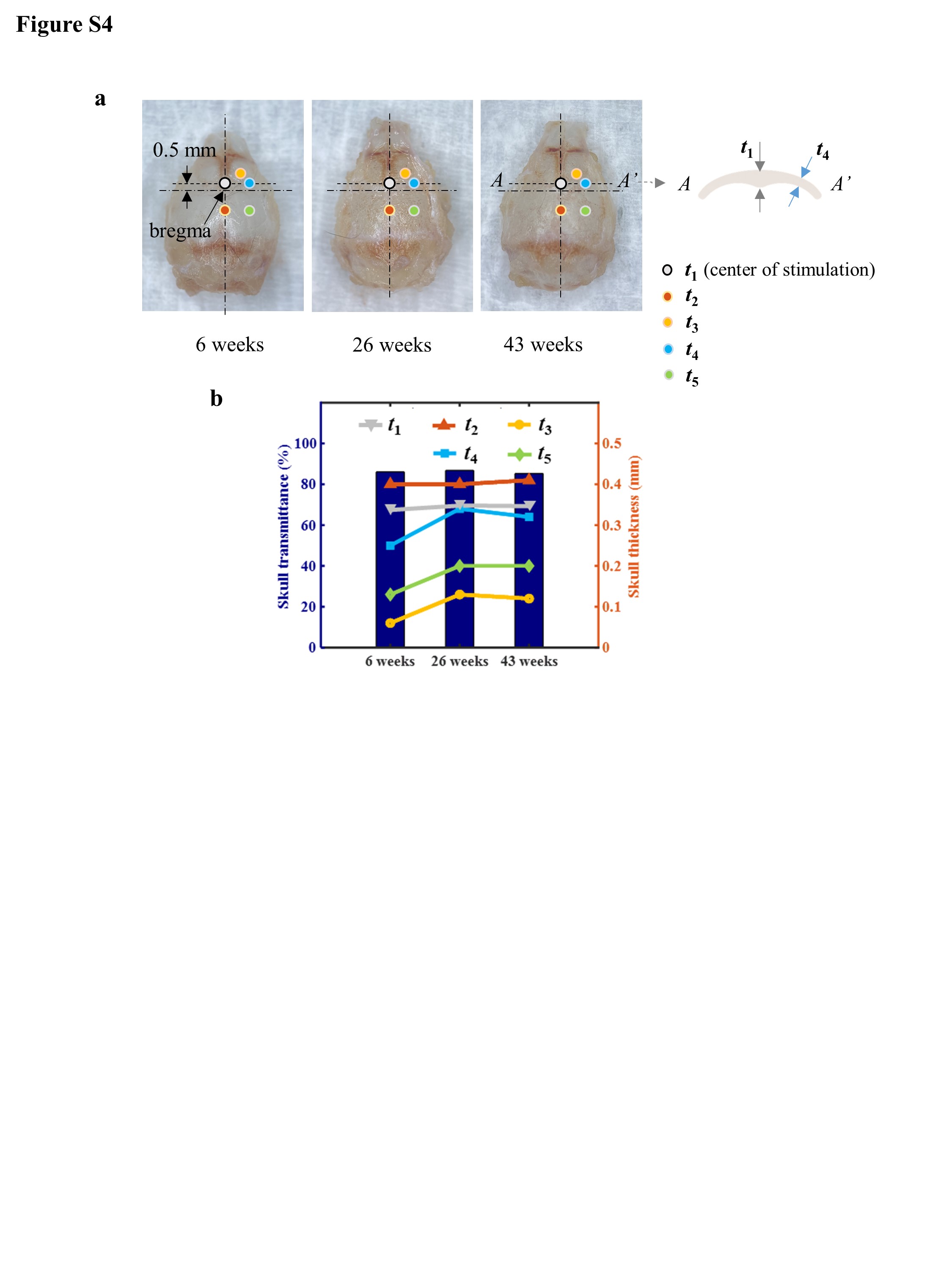

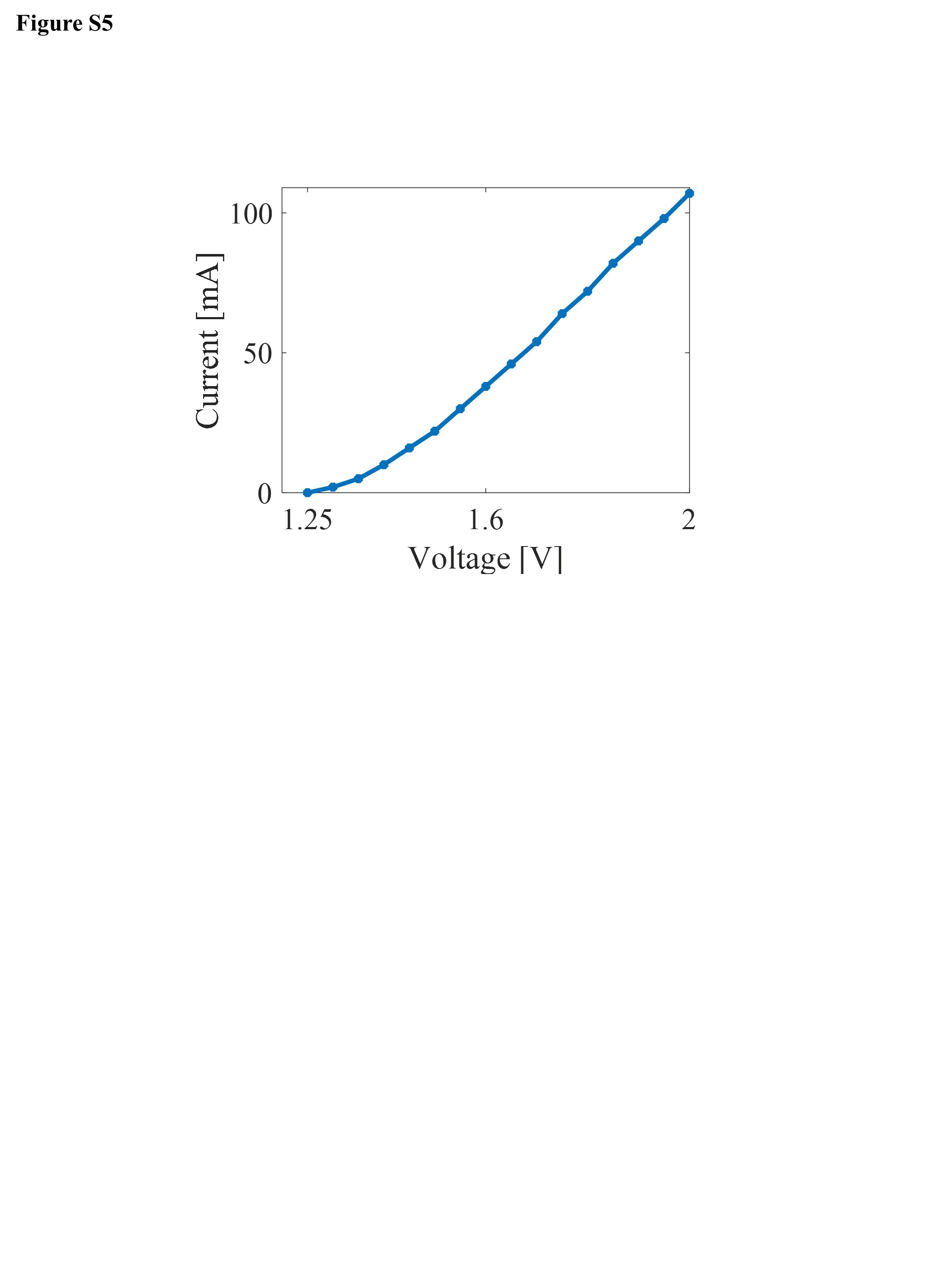


**Figure S5.** *I*-*V* curve of 850 nm µLED.

**Figure S6.** Fabrication process of the hybrid electro-optical stimulator based on PLA MNA. Detailed descriptions of each step are provided in the Materials and Methods. Abbreviations: Polydimethylsiloxane (PDMS), Polylactide (PLA), Indium tin oxide (ITO). Flexible printed circuit board (FPCB) and Micro-light-emitting diode (μLED).


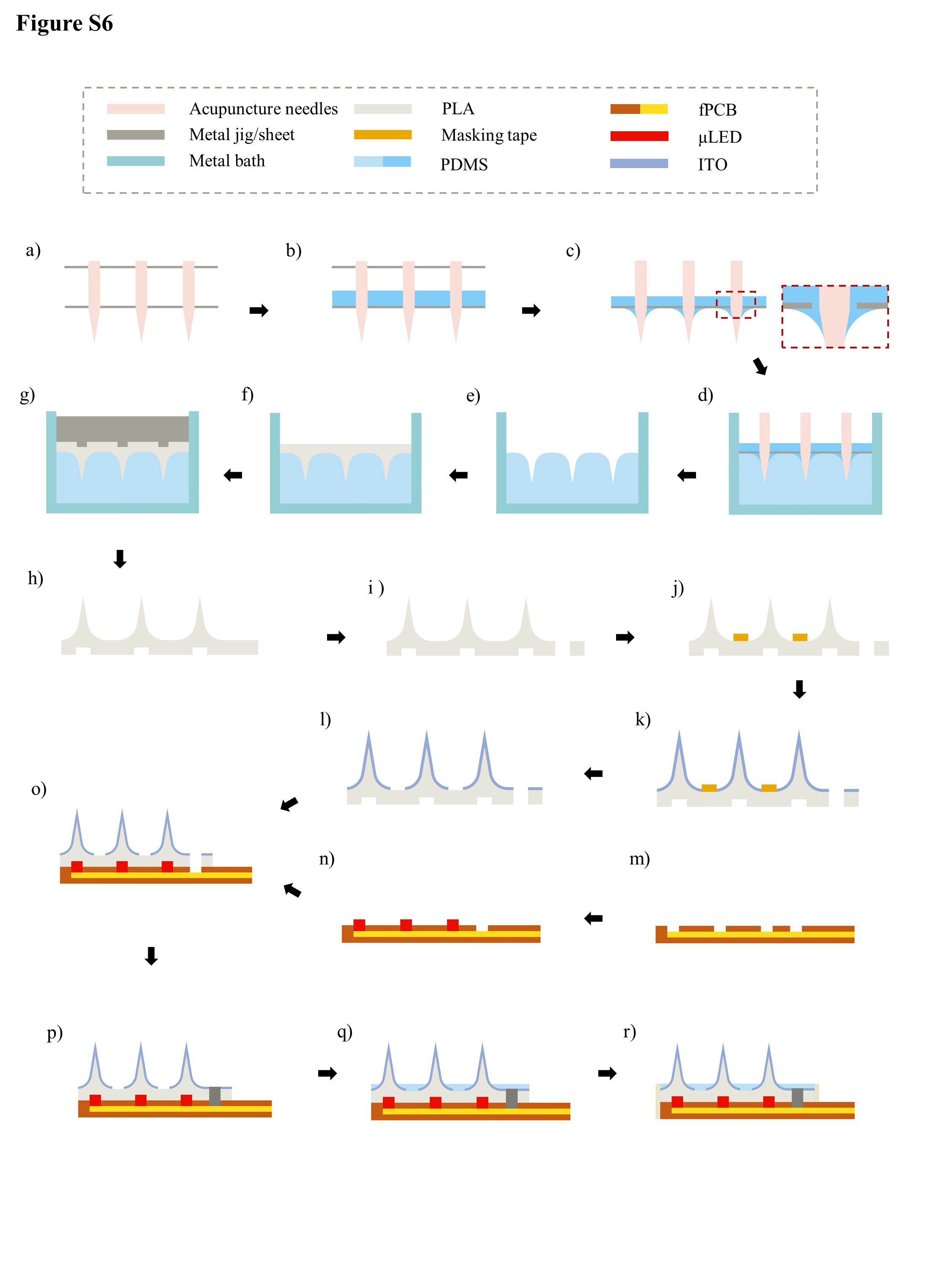


**Figure S7.** Microscopic images captured during MNA fabrication. Perforated metal sheets (a) before and (b) after fixation with an acupuncture needle. (c) PDMS-negative mold and (d) PLA MNA produced after double-casting. The inset shows a magnified view of a single microneedle with an underlying μLED pocket. (e) MNA masked by patterned polyimide tape to electrically separate the center needle and surrounding needles for (f) ITO coating.


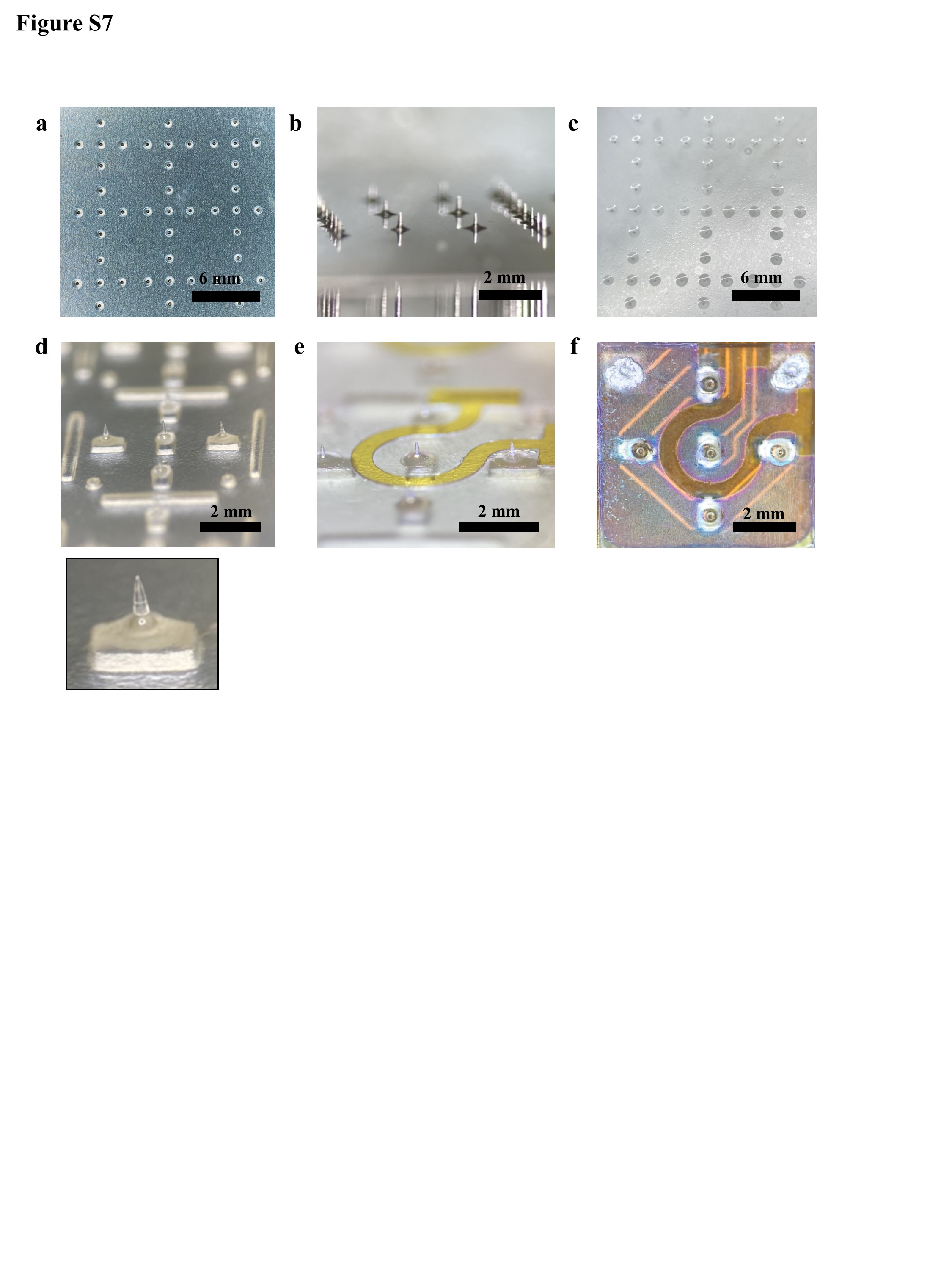

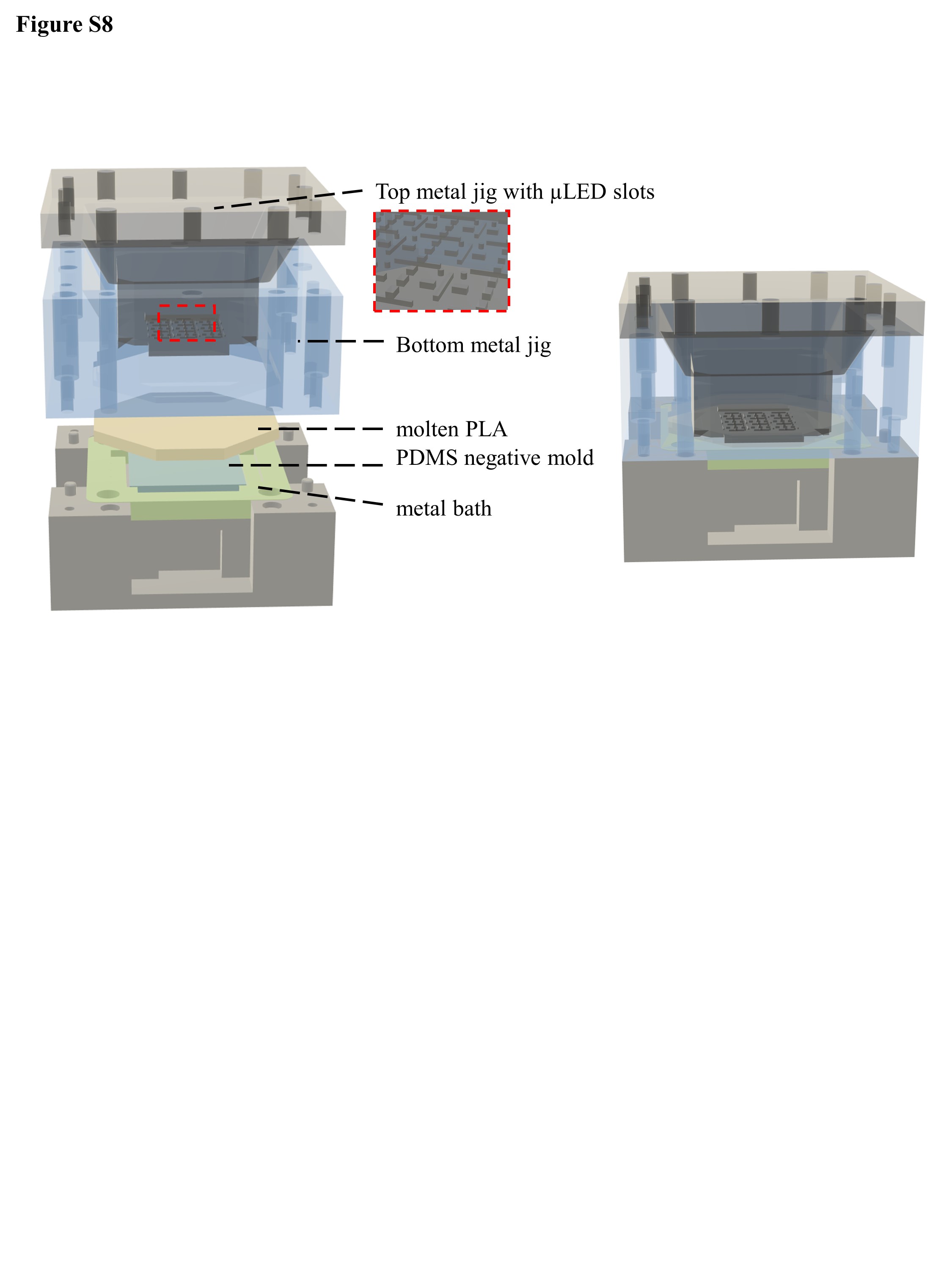


**Figure S8.** A 3D CAD drawing of the aligning metal jig for casting PLA MNA with precise alignment between PDMS-negative mold and top metal jig with µLED slots. Abbreviations: Polydimethylsiloxane (PDMS), Polylactide (PLA), Microlight-emitting diode (μLED).


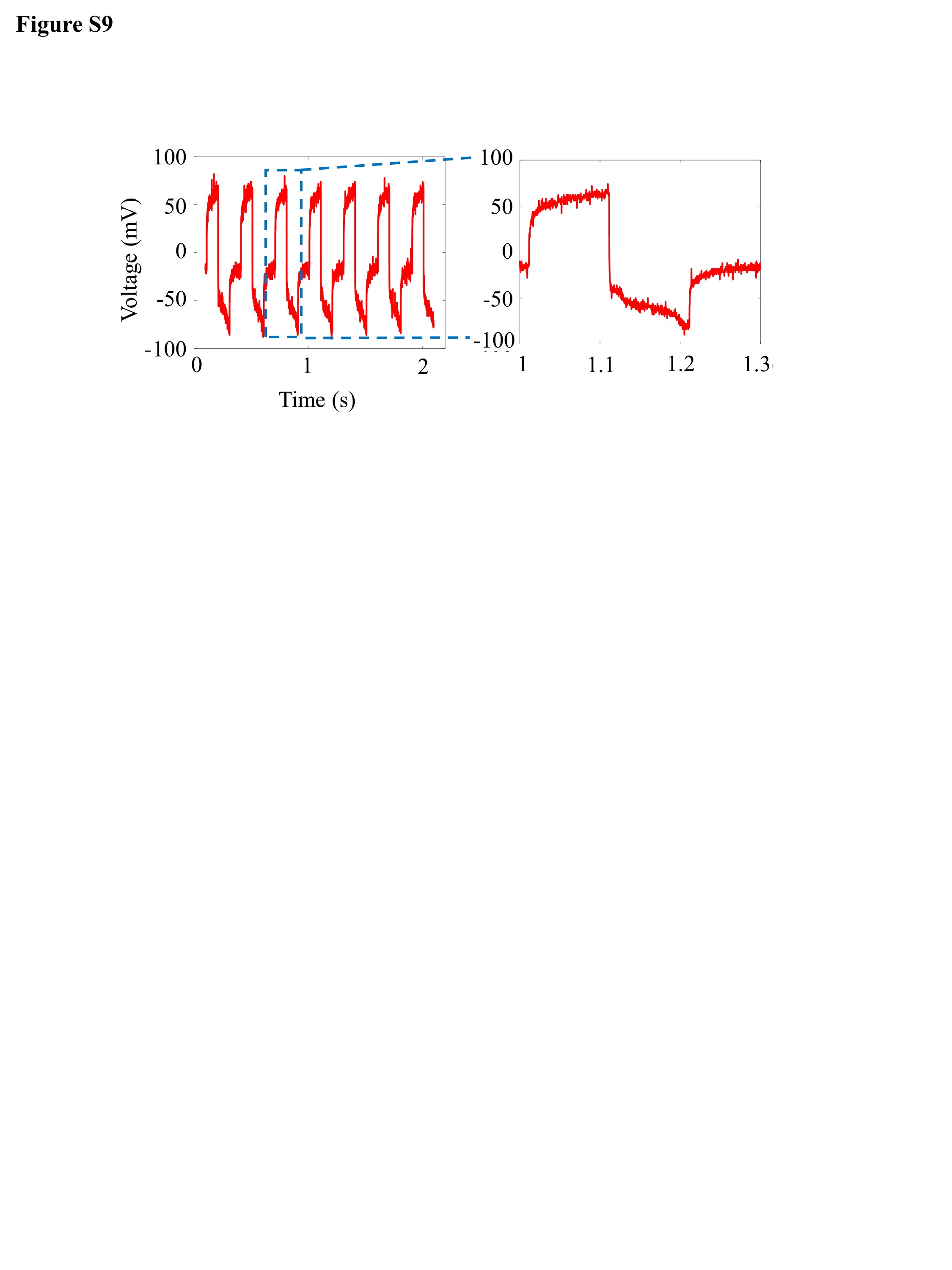


**Figure S9.** Voltage transient of ITO-coated microneedles during current injection into PBS, with an amplitude of 100 µA and a pulse duration of 300 µs, confirming the electrical simulation capability of the ITO coating.


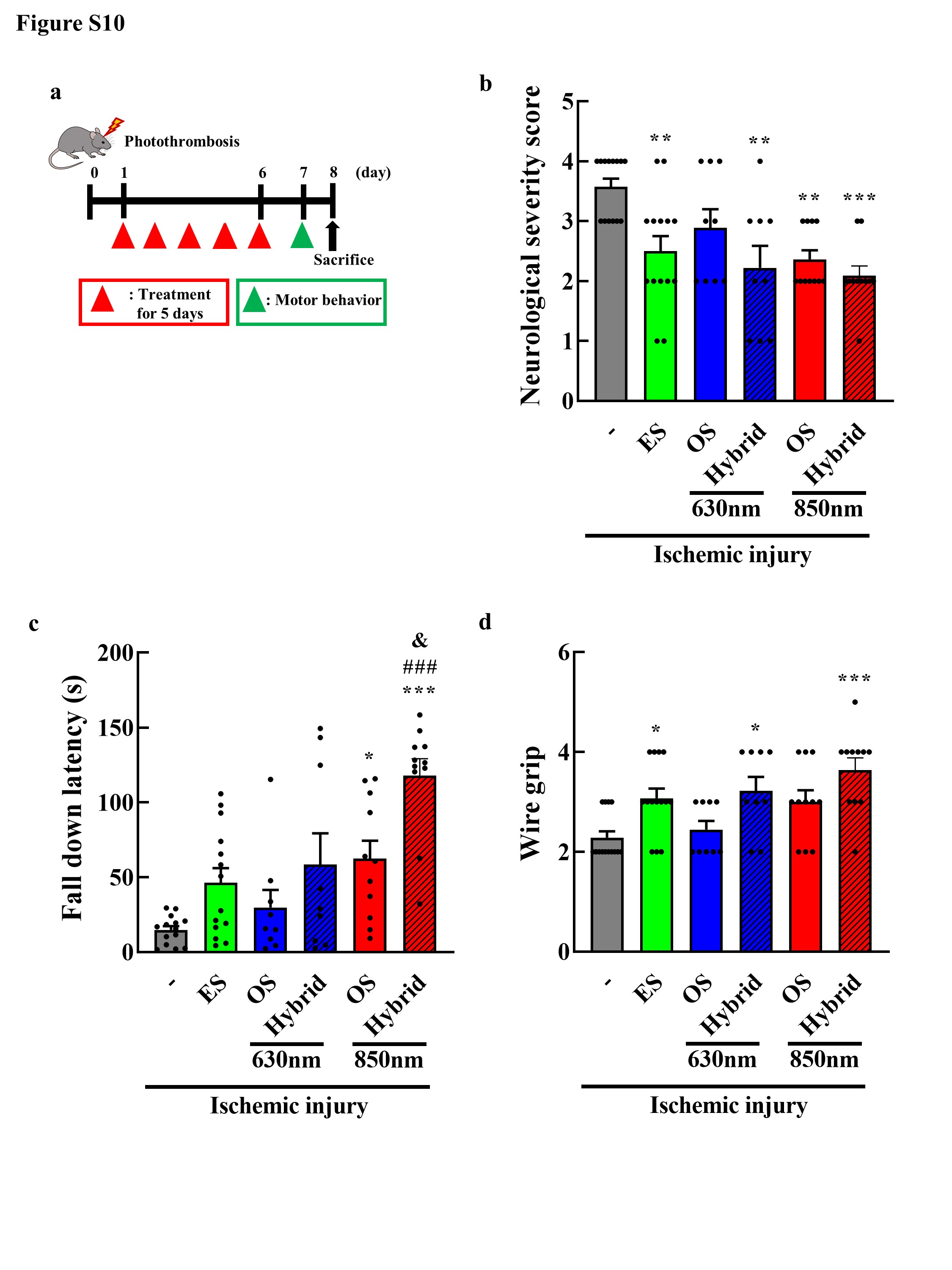


**Figure S10.** Two wavelengths of optical stimulation (630 and 850 nm) were selected to assess the most effective hybrid stimulation for stroke treatment using a photothrombotic cortical ischemia mouse model. (a) Experimental design. The mice underwent behavioral tests to evaluate the recovery of (b) neurological deficits and motor function using (c) rotarod and (d) wire grip tests 7 days after ischemic injury (N = 14 for Vehicle and ES group, N = 9 for OS (630 nm) and Hybrid (630 nm) groups, N = 11 for OS (850 nm) and Hybrid (850 nm) groups). All data are represented as mean ± SEM. Statistical signiﬁcance was determined by one-way ANOVA with Tukey’s post-hoc test. *P < 0.05, **P < 0.01, and ***P < 0.001 versus Ischemic injury group. ###P < 0.001 versus the ES group, and P < 0.05 versus the OS (850 nm) group. Abbreviations: Electrical stimulation (ES), Optical stimulation (OS).


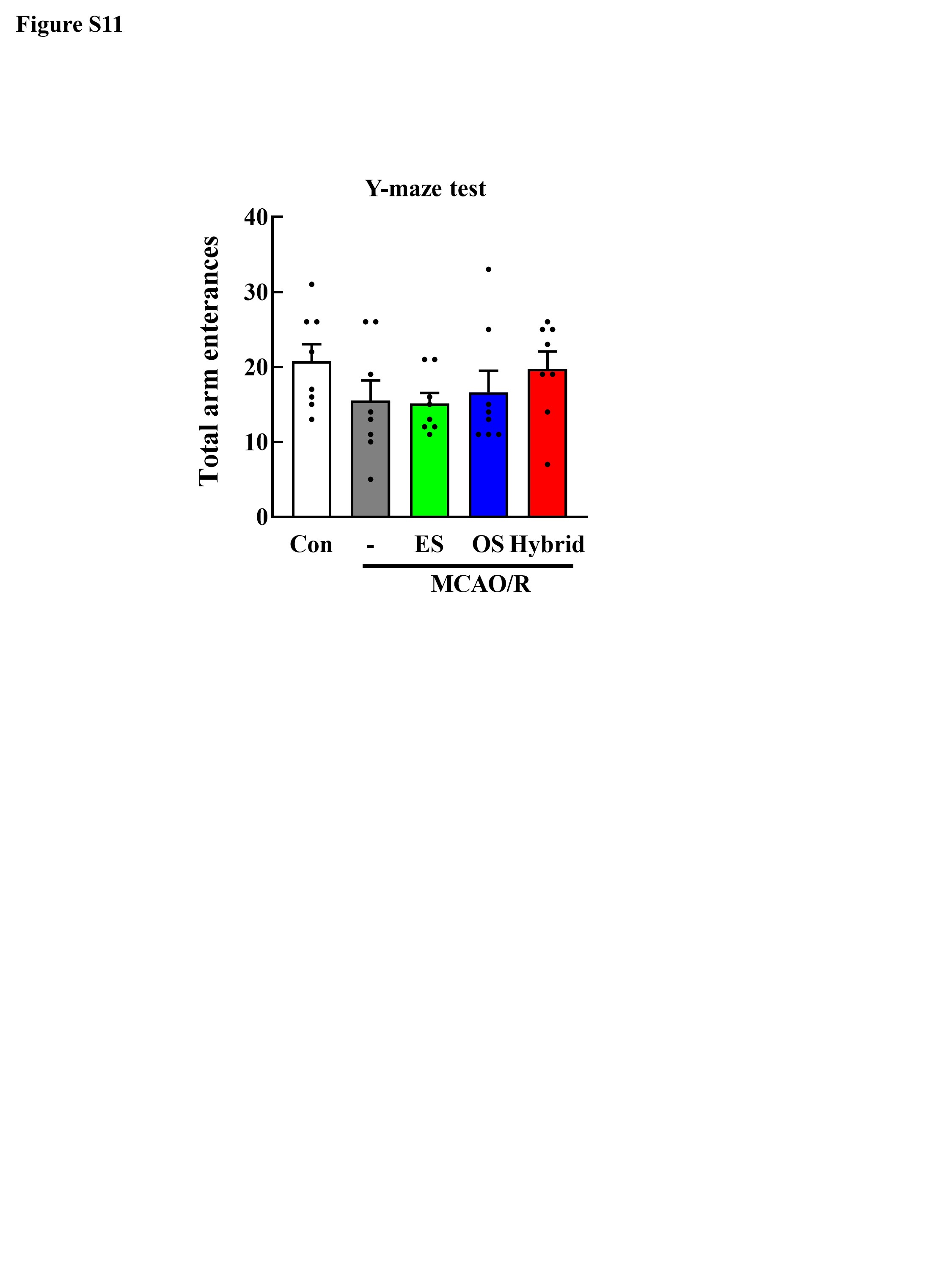


**Figure S11.** Total arm entries in the Y-maze (N = 8 per group).


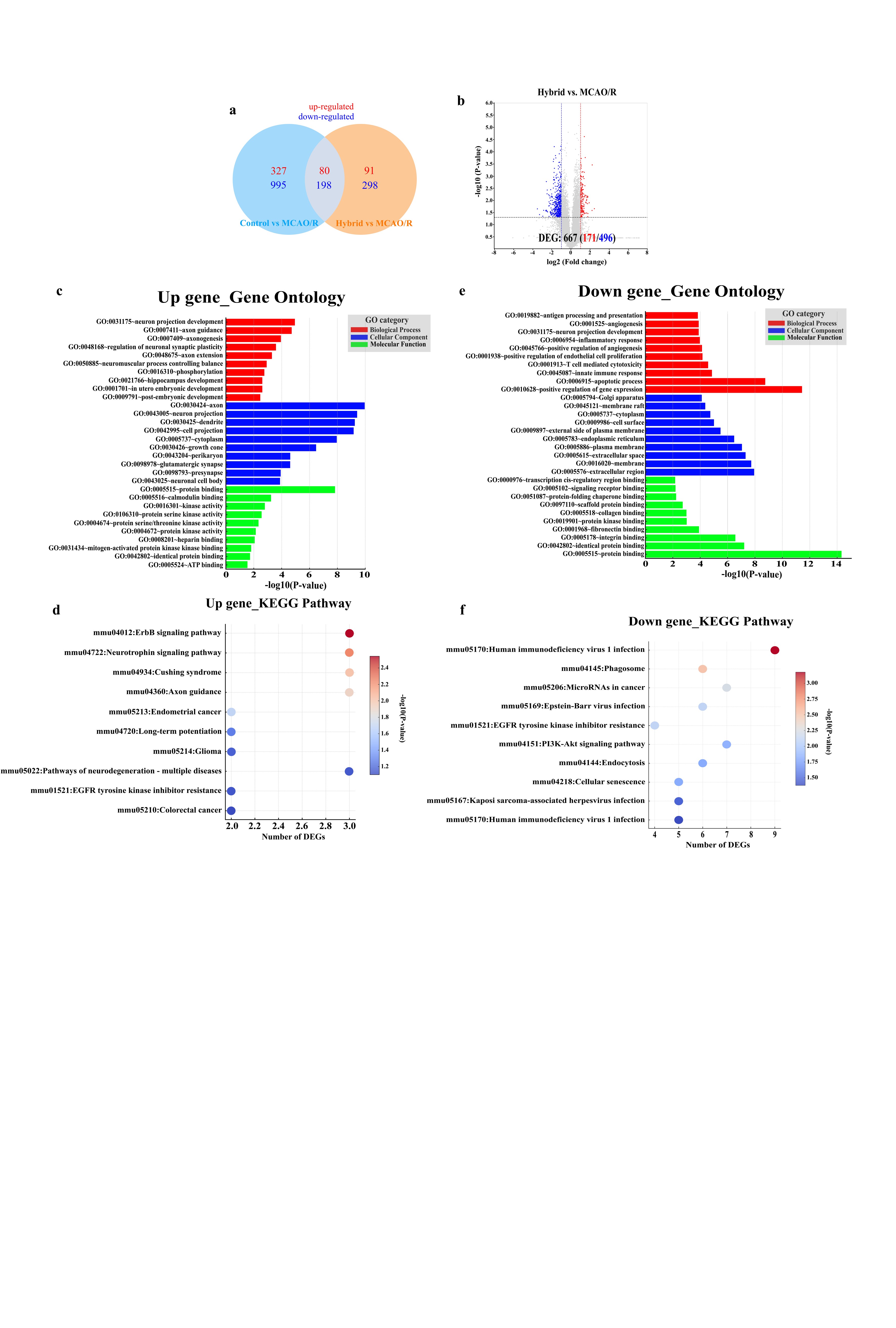


**Figure S12.** GO and KEGG pathway analysis of DEGs following hybrid electro-optical stimulation in MCAO/R. **(a)** Venn diagram showing the number of upregulated and downregulated genes identified in two comparisons: Control vs. MCAO/R and Hybrid vs. MCAO/R. (b) A total of 667 DEGs (171 upregulated and 496 downregulated) were identified in the hybrid vs. MCAO/R comparison, illustrating the specific gene expression changes induced by hybrid electro-optical stimulation using volcano plot. Enriched GO terms are categorized into Biological Processes, Cellular Components, and Molecular Functions for (c) up- and (e) downregulated genes in the hybrid vs. MCAO/R group. KEGG pathway enrichment analysis was conducted to understand the functional significance of (d) up- and (f) downregulated genes in the hybrid vs. MCAO/R group. N = 4 each group. Abbreviations: Middle cerebral artery occlusion/reperfusion (MCAO/R), differentially expressed gene (DEG), Kyoto Encyclopedia of Genes and Genomes (KEGG).


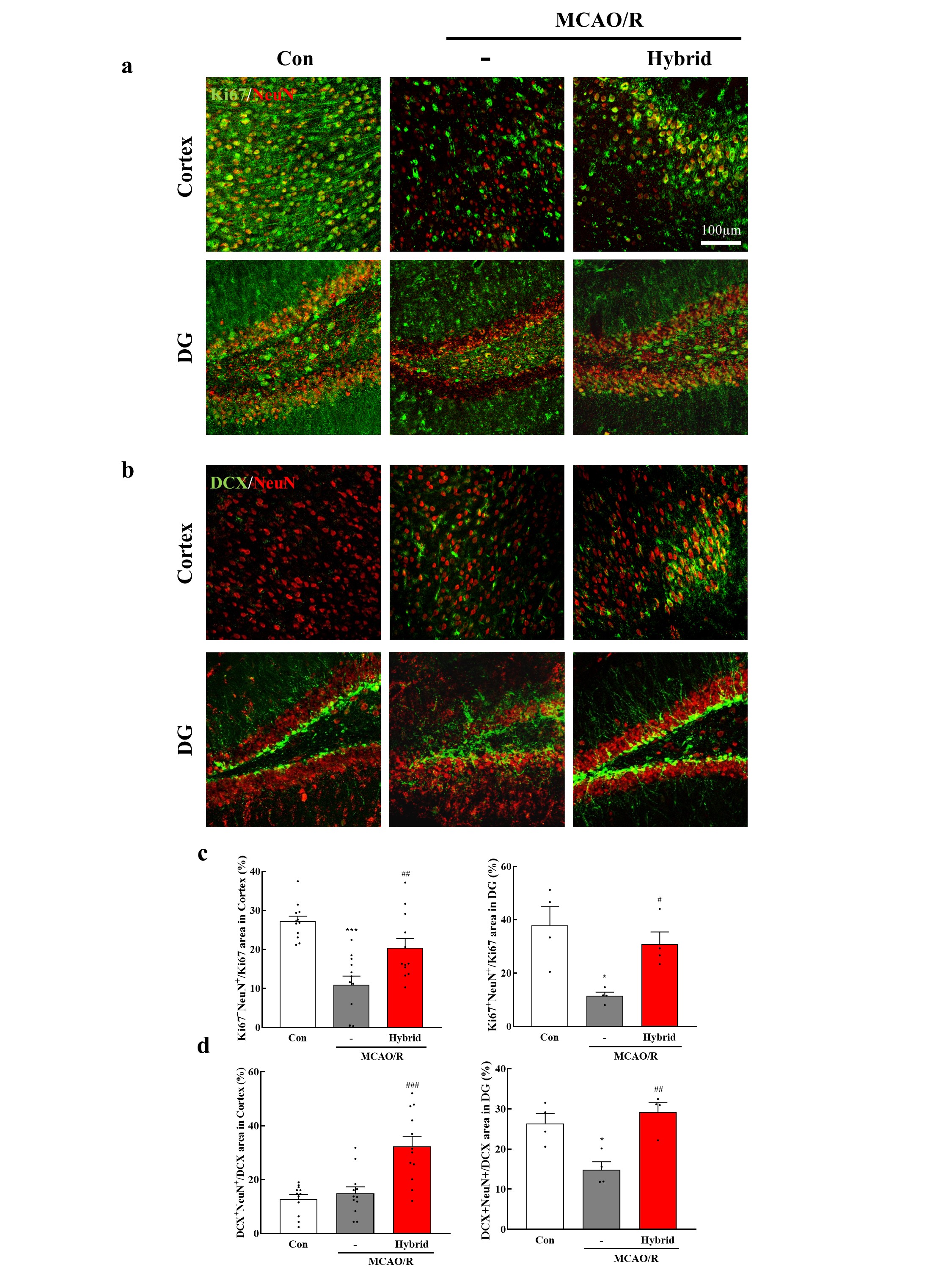


**Figure S13.** Neurogenesis and cell Proliferation in cortex and DG in hybrid electro-optical stimulation mice after MCAO/R. Immunofluorescence staining for (a) Ki67 (green; proliferation marker) + NeuN (red; mature neuronal marker) and (b) DCX (green; neuronal precursor marker) and NeuN (red) in the cortex (N = 12 images each group) and DG (N = 4 images each group). Quantification of (b) Ki67+NeuN+ and (c) DCX+NeuN+ co-localized areas (%). All data are represented as mean ± SEM. Statistical signiﬁcance was determined by one-way ANOVA with Tukey post hoc test. *P < 0.05 and ***P < 0.001 versus Control group. #P < 0.05, ##P < 0.01, and ###P < 0.001 versus MCAO/R group. Abbreviations: Middle cerebral artery occlusion/reperfusion (MCAO/R), and Dentate Gyrus (DG).

**Figure S14.** Effects of electrical stimulation (ES), optical stimulation (OS), and hybrid stimulation on AQP4 expression and polarization in MCAO/R mouse model. (a) Representative images of AQP4/GFAP immunostaining in the peri-infarct cortex 7 days after MCAO/R. (b) Quantification of the AQP4-positive area (N = 9 images per group) and perivascular AQP4 polarization (N = 30 vessels per group) in each group. Data are presented as mean ± SEM. Statistical signiﬁcance was determined by one-way ANOVA with Tukey’s post-hoc test. **P < 0.01 and ***P < 0.001 versus control group. ###P < 0.001 versus MCAO/R group. $$$P < 0.001 versus ES group. &&P < 0.01 versus OS group. Abbreviations: Control (Con), Middle cerebral artery occlusion/reperfusion (MCAO/R), Aquaporin-4 (AQP4), Glial fibrillary acidic protein (GFAP), electrical stimulation (ES), optical stimulation (OS).


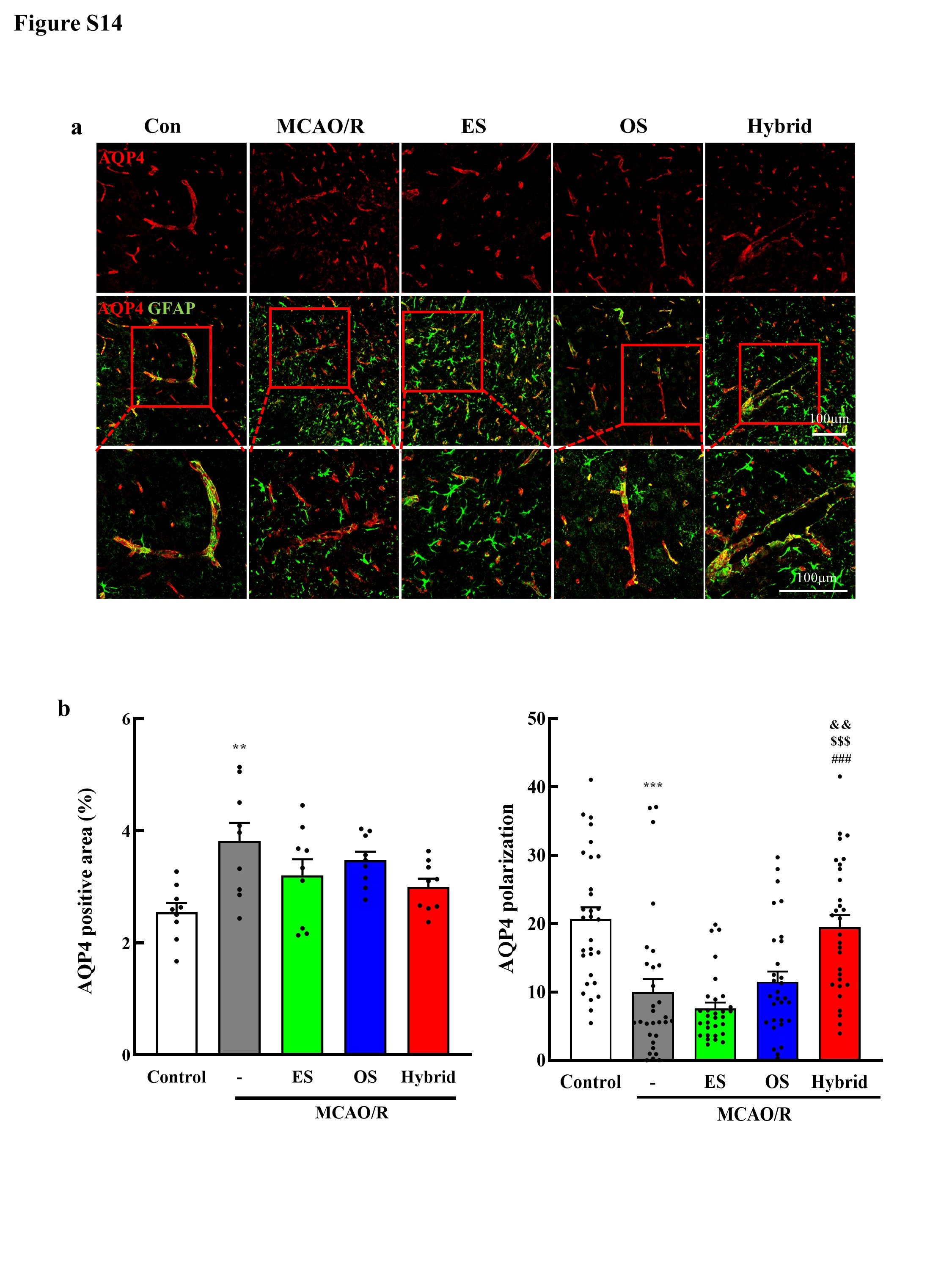


**Figure S15.** Flow diagram of AQP4 polarization. Systemic image analysis of AQP4 polarization. Abbreviations: Fluorescence intensity (FI), Perivascular space (PVS), Region of interest (ROI).


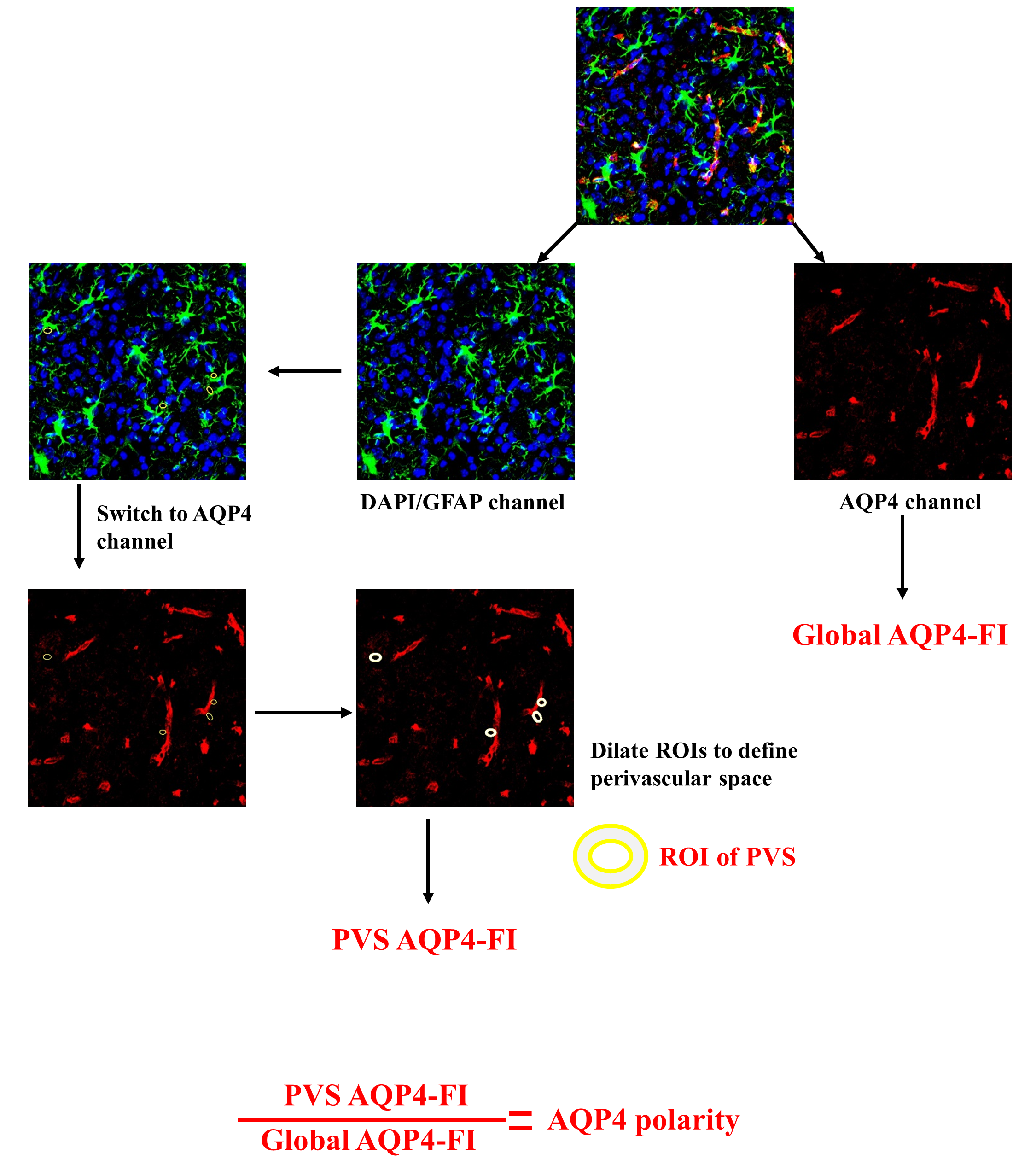

Supplement: Supplementary file 1 — Supporting Information [file ADVS-12-2417449-s001.docx]
